# Soft Tissue and Bone Sarcoma Group, 25th Anniversary Meeting on Sarcomas, April 5–7, 2001, Aarhus, Denmark

**DOI:** 10.1080/135771401200XXXXX

**Published:** 2001-09

**Authors:** John Gibbs

**Affiliations:** Division of Surgery Roswell Park Cancer Institute Elm and Carlton Streets Buffalo NY 14263 USA


*Sarcoma* (2001) **5**, 157–184

### EORTC ABSTRACTS

### Soft Tissue and Bone Sarcoma Group, 25th Anniversary Meeting on Sarcomas, April 5–7, 2001, Aarhus, Denmark

The Soft Tissue and Bone Sarcoma Group of EORTC was founded in 1976 and has since developed into one of the leading cooperative groups in the research of sarcomas and has members from 40 institutions in 14 countries. So far it has conducted more than 40 clinical trials with more that 250 patients included per year. In addition a database with more than 2500 patients has been developed.
The activities of the group have primarily been within the areas of standards for local as well as systemic treatment strategies, new drug development and quality control procedures. The group has an extensive quality control programme involving a strict membership policy, central reviews of responses and pathology, use of a systemic therapy checklist and on-site monitoring of studies.
At the 25th Anniversary Meeting attention was focused on the future of sarcomas with special attention to new developments in the field of molecular biology and cytogenetics, imaging as well as local and systemic treatment. Posters covering all areas of sarcomas were presented.
Unfortunately one of our most loyal members over many years, Alberto Azzarelli, died on 22, February 2001 after several months of suffering. He was not only an important member of the scientific committee of this meeting but for many years a very active and inspiring member of EORTC STBSG and his entire professional life was dedicated to sarcomas. The entire group misses Alberto, but we hope, that in his spirit, we continue the effort to improve the care of patients with sarcomas and therefore we dedicate the 25th Anniversary Meeting to his memory.
We were very pleased that 240 participants joined the meeting and that 85 abstracts were submitted—many more than anticipated—clearly demonstrating the increasing interest in the field.
In general the meeting was very successful and the following abstracts were presented.

*Ole Steen Nielsen, Chairman*

### SCIENTIFIC PROGRAM

### THURSDAY 5 APRIL 2001

8.00–9.00 Registration and poster mounting

9.00–9.15 **Welcome and Introduction:** The past 25-year of EORTC Soft Tissue and Bone Sarcoma Group. *(SI)*
*A.T. van Oosterom, Leuven*

9.15–10.40 **Trial design in sarcomas.**
*Chairmen: F. van Coevorden & O.M. Jensen*

9.15–9.40 Future clinical trial design of sarcoma studies? *(S2)*
*J. Verweij, Rotterdam*

9.40–10.05 How should we measure treatment effects: Progression-free survival as the primary endpoint? *(S 3)*
*M. van Glabbeke, Bruxelles*

10.05–10.30 Evaluation and presentation of clinical trial data on sarcomas. *(S 4)*
*I. Judson, London*

10.30–11.00 Coffee break.

11.00–12.30 **How will molecular biology and cytogenetics help the sarcoma clinicians?**
*Chairmen: Pancras Hogendoorn & S. Daugaard*

11.00–11.30 Molecular biology and cytogenetics in diagnosis of sarcomas. *(S 5)*
*C. Cooper, London*

11.30–12.00 Any role left for sarcoma pathology? *(S 6)*
*C.D.M. Fletcher, Boston*

12.00–12.30 What difference will molecular biology and cytogenetics make to future trials and treatments? *(S 7)*
*L. Helman, Bethesda*

12.30–14.00 Lunch and **Poster viewing**.

14.00–15.00 **What is the best strategy for imaging of sarcomas?**
*Chairmen: A.G. Jurik & L. Svancorova*

14.00–14.20 MR imaging of sarcomas? *(S 8)*
*A.M. De Schepper, Edegem*

14.20–14.40 Future role of PET in diagnostics and response evaluation of sarcomas? *(S 9)*
*S. Stroobants, Leuven*

14.40–15.0 Local and distant staging of bone and soft tissue sarcomas—How good are we? *(S10)*
*A. Saiffuddin, Middlesex*

15.05–16.05 **Future role of local treatment of soft tissue sarcomas**.
*Chairmen: A.N. van Geel & J. Keller*

Correspondence to: John Gibbs, M.D., Division of Surgery, Roswell Park Cancer Institute, Elm and Carlton Streets, Buffalo, NY 14263, USA. Tel: (+1) 716 845 5807; Fax: (+1) 716 845 3434, E-mail: john.gibbs@roswellpark.org

1357–714X print/1369–1643 online/01/030157–28 © 2001 Taylor & Francis Ltd
DOI: 10.1080/135771401200XXXXX

15.05–15.25 Which surgical questions need to be answered in future studies? *(S 11)*
*R.J. Grimer, Birmingham*

15.25–15.45 Which questions in radiotherapy need to be answered in future studies? *(S 12)*
*B. O'Sullivan, Toronto*

15.45–16.05 Is consensus possible? General discussion.
*P. Hohenberger, Berlin*

16.05–16.30 Coffee break.

16.30–17.30 **Potential role of new treatment options for locally advanced soft tissue sarcomas?**
*Chairmen: H.J. Hoekstra & S. Bonvalot*

16.30–16.50 Any role of TNF-perfusion outside the few dedicated centres? *(S 13)*
*F. Lejeune, Lausanne*

16.50–17.10 Prospective role of chemotherapy combined with hyperthermia in sarcomas? *(S 14)*
*R. Issels, Munich*

17.10–17.30 Why have these new local treatments not become more widespread? General discussion. *(S 15)*
*M.H. Robinson, Sheffield*

18.45–20.00 **SOCIAL PROGRAM:** Guided bus tour to 'The Old Town' of Aarhus.

20.00–23.30 **Conference Dinner:** Restaurant Prins Ferdinand

**FRIDAY 6 APRIL 2001**

9.00–10.30 **What should be the future strategy for adjuvant chemotherapy in soft tissue sarcomas?**
*Chairmen: W.P. Steward & P. Woll*

9.00–9.10 Adjuvant chemotherapy has no effect on survival—the EORTC experience. *(S 16)*
*V. Bramwell, London Ontario*

9.10–9.20 Adjuvant chemotherapy may improve survival—the Italian experience. *(S 17)*
*S. Frustaci, Aviano*

9.20–9.40 Pro adjuvant treatment outside clinical trial. *(S 18)*
*R.S. Benjamin, Houston*

9.40–10.00 Contra adjuvant treatment outside clinical trial. *(S 19)*
*K. Antman, New York*

10.00–10.15 How to proceed?
*V. Bramwell, London Ontario*

10.15–10.30 General discussion.

10.30–11.00 Coffee break.

11.00–12.00 **Systemic treatment of advanced soft tissue sarcomas.**
*Chairmen: P. Reichardt & J.Y. Blay*

11.00–11.20 Is Doxorubicin really the standard treatment of advanced soft tissue sarcomas? *(S 20)*
*T. Alvegaard, Lund*

11.20–11.40 New drugs for treatment of metastatic soft tissue sarcomas? *(S 21)*
*A. le Cesne, Paris*

11.40–12.00 How to proceed? General discussion.
*P. Reichardt, Berlin; J.Y. Blay, Lyon*

12.00–13.30 Lunch and **Poster viewing.**

13.30–14.30 **Poster discussion**
*Chairmen: J.A. Radford & R.B. Keus*

14.30–15.30 **What is the optimal treatment of the Ewing/PNET family of tumours?**
*Chairmen: I. Judson & H. Jürgens*

14.30–14.50 The experience of SFOP. *(S 22)*
*O. Oberlin, Paris*

14.50–15.10 What did we learn from the EICESS studies? *(S 23)*
*H. Jürgens, Münster*

15.10–15.30 The SSG/Italian Group strategy. *(S 24)*
*P. Picci, Bologna*

15.30–15.45 After Euro-Ewing 99—any questions left? General discussion.
*I. Judson, London; H. Jürgens, Münster*

15.45–16.00 **Closing of meeting.**
*O.S. Nielsen, Aarhus*

16.00–16.30 Coffee break. **End of scientific meeting.**

16.30–17.30 **Subcommittee Meetings of EORTC STBSG:**

17.30–18.30 **Business meeting of STBSG.**

18.30–20.15 **General meeting of STBSG.**

21.00– Dinner for members of EORTC STBSG. Restaurant 'Queens Garden'.

**SATURDAY 7 APRIL 2001**

09.00–13.00 **General meeting of STBSG** (cont.).
incl. coffee break

13.00–14.00 Lunch. End of Meeting

### Abstracts—Invited Speakers

### The past 25 years of EORTC soft tissue and bone sarcoma group

A. VAN OOSTEROM
*(Department of Oncology, U.Z. Gasthuisberg, KU Leuven, Leuven)*

Preliminary meetings in April—to June 1976—between founding members led to the full development of a protocol in which CyVADic was compared with CyV-Adic in advanced soft tissue sarcoma. This protocol was approved by the EORTC Protocol Review Committee and the Group received the number 62 and the protocol 62761.
The first and founding meeting of the Group was held in Zurich (Switzerland) in September 1976 where G. Bonadonna (Milan—Italy) was elected chairman and H.M. Pinedo (Utrecht—The Netherlands), the driving force, became secretary. The protocol was initiated and in October the first patient had been registered. Pinedo was the study co-ordinator.
Other founding members came from France, Germany, Belgium, Switzerland, the UK and the Netherlands.
The objectives of the Group were to develop, stimulate and co-ordinate studies on all aspects of the treatment of soft tissue sarcomas within the framework of the European Organisation for Research and Treatment of Cancer. The second objective was to organise congresses, symposia and conferences to promote these studies.
The first adjuvant study in which after optimal local treatment the patients were randomised to no treatment or adjuvant CyVADic, started late 1977 and was co-ordinated by V. Branwell (Manchester—UK).

In 1978 the soft tissue sarcoma group merged with the international osteo-sarcoma working party to form the EORTC Soft Tissue and Bone Sarcoma Group (STBSG).
The emphasis in the studies has always remained the treatment in first and second line of advanced soft tissue sarcoma patients. It has led to a priceless collection of data in Brussels of over 3.000 patients stored under the careful guidance of R. Sylvester and since 1989 of M. Van Glabbeke, the Group's statisticians.
Next to the study of many new agents, the Group has developed very strict quality control procedures and played a major role in the development of the RECIST criteria.
In the recent past studies in Ewing sarcoma and several subtypes of soft tissue sarcomas have been initiated. It should be mentioned that the international recognition of this Group has led to a track record of at least one oral presentation at every ASCO and ECCO-ESMO meeting since 1984. Of course major publications, not only in books, but also in the Journal of Clinical Oncology, the European Journal of Cancer, the Annals of Oncology, the British Journal of Cancer and the Journal of the National Cancer Institute but also in the New England Journal of Medicine and the Lancet have appeared.
The future looks bright: in view of the fact that the Group plays a pivotal role not only in the clinical development of the new molecular targeted therapies but also performs translational research in its present studies. The number of participating institutions and countries will certainly increase in the next decades.

**Future clinical trial design of soft tissue sarcoma studies**

J. VERWEIJ
*(Rotterdam Cancer Institute and University Hospital, 3075 EA Rotterdam, The Netherlands)*

Until recently most soft tissue sarcomas, due to the relatively low incidence of these diseases, have been treated and studied grouped together as if they were one disease. Retrospective analyses of large databases have however identified certain subtypes that tend to respond differently from others and the increased knowledge should guide us to a better differentiated trial design.
In addition, due to the increased knowledge of molecular biology and the improved molecular diagnostics more and more specific receptors on the cell surface have been identified that can be used as targets for anticancer treatment. Frequently these receptors are specifically overexpressed in specific subtypes of sarcomas and this increasing knowledge should also lead to a more targeted treatment. Inhibitors of these more selective targets are currently in development. Examples are inhibitors of signal transduction, inhibitors of farnesyltransferase and inhibitors of angiogenesis or matrix metallo proteinase. Importantly, many of these latter agents, in *in vitro* and *in vivo* models, mainly exhibit growth inhibition rather than tumour regression. This aspect is also important to take into account when designing a trial. If tumour regression can not be expected, phase II studies seem rather useless. Once time to progression and/or survival become the main endpoint, the phase IIB or preferably phase III trial design becomes the preferred one. Obviously, moving from phase I directly to phase II would involve a giant leap of faith. Intermittent alternative trial designs are therefore currently under consideration.
Whatever the issue, it is clear that in the near future we will more and more witness the design of large trials as well as the design of trials in specific subtypes of sarcomas or even a more general approach of targeting a molecular target regardless of subtype. Examples of these are the potential use of conventional cytotoxics such as Taxol for angiosarcomas or Ifosfamide in synovial sarcomas, but also the more recently developed agents, such as STI 571 in C-kit overexpressing sarcomas or agents directly towards the ppar-$\gamma$ ligand in liposarcomas. The specificities of these trial designs will be discussed in detail.

**How should we measure treatment effects: progression free survival as the primary end-point?**

M. VAN GLABBEKE
*(EORTC Data Center, Brussels, Belgium)*

*Introduction:* The aim of this presentation is to explore whether and when we can recommend progression free survival as the principal end-point for phase II trials on soft tissue sarcoma.
*For which agents?* Response to therapy, based on measured decrease in the size of cancer lesions, is the most effective end-point to document biologic anticancer activity of cytoreductive agents and consequently to identify potential new cytoreductive drugs. The RECIST criteria provide an harmonized method of response evaluation. For non cytoreductive anticancer agents, biologic activity is not expected to translate into shrinkage of lesions, but rather in stabilization of progressive disease. The RECIST guidelines recognize that progression free survival and/or time to progression may be a valuable alternative end-point to provide an initial estimate of biologic effect for those agents.
*Statistical designs:* The classical phase II designs (Simon, Fleming, Gehan) are only applicable to phase II trials with a binary primary end-point (success vs failure). Success can be defined as absence of objective progression (as defined by the RECIST criteria) at a fixed selected time point (i.e. 3 or 6 months), if all patients are followed until this point. This is a valid end-point only if disease progression has been documented before starting therapy.
*Target success rates:* Two baseline parameters are needed to compute the sample size and decision rules with those designs: the minimum success rates (i.e. progression free -PF- rate) expected from an active drug (P1) and the maximum success rate expected if the drug is inactive (P0). The success rate observed with the best available regimen for the targeted patient population is generally used as P1. We have estimated relevant PF rates for soft tissue sarcoma trials from the STBSG database.
For non pre-treated patients, PF rates for active regimen were estimated from the data of 1154 patients treated with 1st line anthracycline containing chemotherapy, with an externally reviewed diagnosis of leiomyosarcoma (531), mfh (217), synovial sarcoma (115), liposarcoma (110), fibrosarcoma (68) and neurogenic sarcoma (113). The 3 months estimated PF rates varied from 77% (synovial sarcoma) to 58% (leiomyosarcoma) and the 6 months PF rates from 56% (synovial sarcoma) to 38% (mfh). In 61 leiomyosarcoma from GI origin (now identified as GIST), those figures were 44% and 30% respectively.
For pre-treated patients, PF rates for active regimen were estimated from 124 patients treated with ifosfamide or DTIC, after failure of anthracycline containing regimen. Overall PF rates at 3 and 6 months were estimated to be 44% and 18% respectively. PF rates for inactive regimen were estimated from 253 patients included in 2nd line trials with 9 agents that did not demonstrate activity. In those patients, the overall PF rate at first disease evaluation (6 to 8 weeks after treatment start) was 21%.
*Conclusion:* Progression free rates may be appropriate primary end-points for phase Ii trials with non cytoreductive agents in soft tissue sarcoma, but the patients' selection, follow-up period and parameters of the statistical design need to be adapted.

**Evaluation and presentation of clinical trial data on sarcomas**

I. JUDSON
*(Royal Marsden Hospital, London, UK)*

*The problem:* Soft tissue sarcomas (STS) are a heterogeneous group of disparate diseases. Outcome is determined by disease site, tumour size, grade and histological subtype. Nevertheless, owing to their rarity, and the paucity of effective treatments, it remains common practice to conduct Phase II and Phase III trials of chemotherapy regimens in mixed populations of patients.

Trials of adjuvant chemotherapy provide an example of the difficulty caused by this process. Individually these have generally failed to demonstrate a survival benefit.[1] In part this is due to their small size, in part patient heterogeneity. The meta-analysis reported in 1997[2] aroused controversy owing to the inclusion of studies involving patients with particularly poor prognosis, e.g. uterine sarcoma, and patients with indolent, low grade tumours, and also the use of low dose regimens that would not conform to current ideas of best practice. Overall the results showed a benefit from treatment in terms of disease-free but not overall survival. Nevertheless, the debate served to highlight the results of the Italian Sarcoma Group study reported by Frustaci et al[3] which shows a survival advantage for aggressive chemotherapy in selected patients with extremity and limb girdle high grade tumours. Maturing data from this small trial appear to confirm the early results.

How then are we to judge the results of studies carried out in mixed populations of patients?

*Assessing clinical trial data:* Firstly, it is necessary to look carefully at all the known prognostic factors. Retrospective studies have been conducted using the EORTC Soft Tissue and Bone Sarcoma Group database of over 2,000 patients treated for advanced or metastatic disease with anthracycline-containing chemotherapy. These act as an excellent source of unbiased information. It has been demonstrated that favourable factors for a response to chemotherapy are: absence of liver metastases, young age, high grade and liposarcoma. Similarly, longer survival is associated with: low grade, good performance status, long disease-free interval from diagnosis, young age and absence of liver metastases.[4] In a separate study it was shown that patients with small metastases only in the lung are most likely to respond to chemotherapy.[5] With regard to histological type, apart from the favourable response in the case of liposarcoma, the converse is clearly true for visceral tumours, especially gastrointestinal stromal tumours (GISTs) which rarely, if ever respond to cytotoxic chemotherapy.

In other words, if a Phase II study in advanced STS reports a 60% response rate using a standard chemotherapy combination, it most probably means that the investigators have selected young, fit patients with extremity, high grade tumours, who have lung-only, low volume metastatic disease. If the authors are unwise or unscrupulous they may wish to persuade us that their excellent results are due to their special 'recipe', especially if dose escalation has been used. This sort of bias can only be excluded by performing randomised trials. For example an EORTC Phase II study used GM-CSF to allow escalation of the dose of doxorubicin from 50 to 75 mg/m$^2$ in combination with ifosfamide. An excellent response rate of 45% was obtained.[6] Unfortunately, in a subsequent randomised trial there was no advantage for the higher dose of doxorubicin.[7] Similarly, in a randomised Phase II trial comparing standard doxorubicin versus liposomal doxorubicin (DOXIL®/CAELYX®) both arms performed poorly with response rates of only 9% and 10%.[8] The latter response rate in a single arm study would have resulted in the conclusion that CAELYX® was inactive. Increasingly, our improving understanding of sarcoma biology is resulting in the ability to treat individual tumour types selectively. The demonstration that agonists of the peroxisome proliferator-associated receptor gamma (PPARγ) such as troglitazone, cause differentiation of certain types of liposarcoma offers hope of improved therapy in this disease type.[9] Recently, the introduction of a receptor tyrosine kinase inhibitor for the treatment of GISTs seems set to transform our ability to treat this otherwise refractory disease. The introduction of such new treatments will set the standard for defining the patient population under study, since response will be determined by expression of the appropriate receptor.

Presentation of trial data: In conclusion, in order for your data to be meaningful to others, it is vital to include all the data you would like to see for yourself. All prognostic information needs to be included. Any known bias in patient selection should be transparent, and excessively optimistic claims should be avoided. Wherever appropriate, molecular diagnostic techniques should be used to clarify exactly what population of patients has been treated. The precise methods used to assess treatment benefit need to given in detail. In most cases promising results will require confirmation in Phase III trials.

**References**

1 Tierney JF, *et al. Br J Cancer* 1995; 72:469–475
2 Sarcoma Meta-analysis Collaboration. *Lancet* 1997; 350:1647–1654
3 Frustaci F, *et al. Proc Am Soc Clin Oncol* 1999; 18:546a (abstr. 2108)
4 Van Glabbeke M, *et al. J Clin Oncol* 1999; 17:150–157
5 Van Glabbeke M, *et al. Proc Am Soc Clin Oncol* 1999; 18:542a (abstr. 2093)
6 Steward WP, *et al. Cancer Chemother Pharmacol* 1993; 31 suppl 2 S241–244
7 Le Cesne A, *et al. J Clin Oncol* 2000; 18:2676–2784
8 Judson I, *et al.* Eur J Cancer 2001, *in press.*
9 Demetri G, *et al. Proc Am Soc Clin Oncol* 1999; 18:535a (abstr. 2064)

**Molecular biology and cytogenetics in the diagnosis of sarcomas**

C.S. COOPER, J. CLARK, S. EDWARDS, P. FLOHR, M. JOHN, I. GIDDINGS, K. MAILLARD, R. WOOSTER & A. JACKMAN

*(Institute of Cancer Research, The Haddow Laboratories, Cotswold Road, Sutton, Surrey, UK)*

With the publication of the human genome sequences and the identification of around 40,000 human genes a major challenge that now faces the scientific currently is how to use this information to help cancer patients. One very powerful platform that can be used to achieve this goal is microarray technology that allows many thousands of genes to be examined simultaneously. We have through the setting up of ICR 'spotted microarray facility' used this technology in a number of distinct strategies.

CB30865 is a drug of unknown mechanism of action that has activity against a broad range of tumour cell lines. To investigate the molecular basis of resistance to CB30865 we have used microarray technology to compare gene expression in a tymphoblatoid cell line W1L2 with a variant of this line W1L2:R865 that shows a 300-fold increase in resistance CB30865. cDNA probes prepared from the W1L2 cell line and from the W1L2:R865 cell line were hybridized simultaneously to a microarray of 7,000 clones randomly selected from a cDNA library prepared from the W1L2:R865 cell line. These studies led to the identification of *MSS1* which encodes a component of the 26S Proteosome complex as a gene whose level of RNA expression correlated with drug resistance. Based on these observations a derivative of CB30865 called CB300919 was tested and subsequently discovered to be an inhibited of cellular proteosome activity.

We have also used microarray technology to identify genes that are amplified and overexpressed in human cancer. As a test system initially to determine that the procedure was working used the breast cancer cell line BT474 which contains amplicons at 17q11–21 17q22–23 and 20q13. A microarray of 10,000 clones randomly picked a cDNA library prepared from the BT474 cell line was hybridised n comparative genomic hybridisation (CGH) experiments to DNA from BT474 cells and to control muscle DNA. These studies identified 68 amplified genes from the three chromosome 17 and chromosome 20 amplicons and studies on gene expression were used to identify from these 5 new candidate oncogenes. This procedure has now been applied to several amplicons in human sarcomas and the results will be presented.

Controversy still exists over the correct classification of some groups of sarcomas. Particularly it has been proposed that malignant fibrous histiocytoma may in most cases be reclassified pleomorphic leiomyosarcomas, rhabdomyosarcomas or liposarcomas. We are currently using microarray technology to collect expression profiles of primary sarcomas and using hierarchical clustering to determine the relation of MFH tumour to other tumour groups. Preliminary results of these analyses will be presented. We conclude that microarray technology represents a powerful technique that may be utilised in several distal approaches to aid in the management of human sarcomas. (*We acknowledge the Cancer Research Campaign for their support of this project*)

**Any role left for sarcoma pathology?**

C.D.M. FLETCHER
*(Brigham & Women's Hospital & Harvard Medical School, Boston, USA)*

The title chosen for this talk by the organisers is, of course, provocative and deliberately naïve. There is no question that cytogenetics and molecular genetics have had significant impact on our understanding of mesenchymal neoplasia and, in certain circumstances, on our ability to make objective and more reproducible diagnoses. There is also some limited evidence to suggest that molecular genetic data may provide prognostic information in certain tumour types. The role of gene expression profiling is, as yet, largely unexplored but to date has only validated conventional pathologic subclassification in other types of cancer. However, the notion that these techniques will supplant conventional histopathological analysis within the coming 20–25 years is essentially laughable for the following four major reasons:

1. In simple numerical terms, the majority of soft tissue sarcomas do not show any specific, consistent or currently detectable cytogenetic or molecular genetic abnormality;
2. In light of their morphologic heterogeneity, a substantial proportion of soft tissue sarcomas can only be recognised as such by conventional histopathology—there are no surrogate molecular genetic markers of malignancy;
3. The technologies required to take optimal advantage of molecular genetic/cytogenetic analysis are available in very few centres and are often poorly integrated with clinical need;
4. Cost constraints, the need for appropriately trained staff and the relative rarity of soft tissue sarcomas combine to make it very improbable that most institutions will establish and utilize molecular genetic techniques in the foreseeable future.

The fact remains that conventional pathologic analysis, in particular accurate histologic subtyping and (in some circumstances) grading, provides the most crucial information in terms of diagnosis, prognosis and determination of therapy in soft tissue sarcoma patients. Furthermore, the wide availability of this diagnostic methodology, its exceptional cost effectiveness and the relative ease with which tissue samples (or slides) can be shared with other pathologists (or treatment centres) make it highly unlikely that this approach will be superceded in our lifetimes.

Having said that, molecular genetic approaches will unquestionably bring new information and insights which in, in combination with the constant advances in clinicopathologic assessment of these tumours, will help to refine diagnosis, classification and prognosis, at least in some major academic centres. The two main goals in the coming decades should be a) to facilitate a collaborative/integrated approach between clinical and more basic scientists working on soft tissue sarcomas in pathology/oncology and b) to ensure that as many patients as possible are treated in specialist centres, in which best advantage can be taken of any diagnostic and prognostic advances.

**The impact of molecular genetics on the future management of sarcomas**

L. HELMAN
*(National Cancer Institute, Bethesda, Maryland, USA)*

The increasing application of molecular techniques in sarcomas is having significant impact on our ability to diagnose, make prognostic predictions and treat a variety of sarcomas. The discovery of recurrent reciprocal translocations in a variety of sarcomas and the subsequent cloning of the resultant fusion genes have fueled a major change in the diagnostic criteria for such tumors. The ease and widespread application of RT-PCR enables pathologists to confirm or question diagnoses based on molecular criteria. Furthermore, translocation-derived fusion proteins have recently become the target of immunotherapy approaches. Specific technological advances are also beginning to profoundly affect the management of sarcomas. The increasing ease of sequencing tumor DNA has led to the identification of specific point mutations in genes leading to alter signaling of the tumor cells. This finding, in turn, has led to a widespread effort to develop drugs specifically targeting the! se altered signaling pathways. A recent example of the success of this approach is the use of STI 571 in treating GIST tumors, where the drug specifically targets the alteration in the c-Kit gene seen in these tumors. More recently, the application of expression profiling or array technology to sarcomas has the potential to further refine the classification of these tumors and identify new potential therapeutic targets. We are just beginning to see the potential power of high-throughput SNP technology to identify risk categories and critical signaling pathways in these tumors. Illustrations of these approaches and their effects on clinical management of specific tumors will be discussed.

**Medical imaging of soft tissue sarcomas**

A.M.A. DE SCHEPPER, L.H.L. DE BEUCKELEER, X. WANG & J. GIELEN
*(University Hospital Antwerp, Belgium)*

We will present the ability of magnetic resonance imaging in staging, grading, tissue characterization, percutaneous biopsy, and post therapeutic surveillance of soft tissue tumours. Well known staging parameters such as extent, relationship with adjacent structures, and detection of intralesional necrosis are used in the MR protocol for locoregional staging. Bone scintigraphy and high resolution CT scan of the lungs are best methods for ruling out metastatic spread. A variety of (solitary or combinations of) grading parameters is described in radiological literature. The role of MR imaging is to afford recognition of these lesions that need further aggressive work-up, excluding all others. Despite controversial reports, the definite role of MR imaging in grading of soft tissue tumours seems to become established. As for grading, a lot of individual parameters used for tissue characterization have low sensitivity, but combinations of these parameters (age, site, signal intensities, ...) are more useful and often allow to predict a specific diagnosis or to narrow down the list of differential diagnosis. Local recurrences of soft tissue tumours are frequent and can be detected accurately by an 'easy-to-use' MR algorithm. A comparative study between MRI and 'whole specimen' histopathology proves that the MR signal of different tumour components can easily be explained by their different histological composition but that the creation of MR prototypes of various soft tissue tumours is illusory because of tumour components may be intermingled, change in time and sometimes have adverse effects on the overall MR signal intensity. Moreover cellularity, extent of extracellular spaces and relationship between nuclear and cytoplasmatic size will influence signal intensity on different pulse sequences. The value and risks of percutaneous biopsy will shortly be highlighted.

**Future role of PET in diagnostics and response evaluation of sarcomas?**

S. STROOBANTS
*(Department of Nuclear Medicine, U.Z. Gasthuisberg, KU Leuven, Leuven)*

Over the last years, positron emission tomography (PET) has gained widespread enthusiasm as an innovative technique in oncology. In contrast to conventional imaging techniques, such as computed tomography (CT), ultrasound (US), and magnetic resonance imaging (MRI), which are based on differences in *density* of tissues, PET relies on the differences in *metabolism* of cancer cells. For instance, most tumour cells have a higher rate of glycolysis in comparison with non-neoplastic cells. This change in carbohydrate metabolism can be visualized with PET using the radioactive glucose analogue called fluorodeoxyglucose (FDG). PET has the possibility of imaging the whole body within one image session (frequently used in staging procedures) and is able the quantify changes in metabolism, a parameter of use in evaluating the aggressiveness of the tumour or assessing response to treatment.

Although the experience of PET in oncology is growing fast, data on the value of PET in the management of sarcomas is limited and mainly focusing on the ability of FDG-PET in ***differentiating benign from malignant*** (mostly soft tissue) masses. FDG uptake in high-grade sarcoma proved to be significantly higher than those of benign or low-grade tumours, although there was a substantial overlap between the groups. Also the pattern of FDG within the mass seemed ***related to the histopathologic grade:*** very heterogeneous in high-grade and uniform or absent in the low-grade ones. Further insight in the association between FDG uptake and other pathologic features was given by Folpe *et al.*[1] who found that FDG uptake was associated not only with histopathological grade but also with cellularity, mitotic activity, MIB labeling index, and p53 overexpression. Since clinical management is depending on histopathologic grading and sarcomas are heterogeneous neoplasms, FDG-PET may help ensure accurate grading by guiding biopsy toward the most biologically significant regions of large masses. Further follow-up will be necessary to determine whether FDG-PET provides independent prognostic information.

Data on the value of FDG-PET in ***detection of disease extend*** is limited. Lucas *et al.*[2] evaluated the capacity to identify local recurrence (compared to MRI) and pulmonary metastases (compared to CT) in 62 patients with soft-tissue tumours after treatment. For the detection of local disease, MRI proved to be more accurate than PET whereas for the identification of lung metastases, CT proved to be the most sensitive and PET the most specific technique. The major advantage of PET was its ability to detect other metastases at unexpected sites.

Finally, PET can be used to ***evaluate the effect of treatment***. Since metabolic changes precede the morphological ones, PET seems a promising tool to discriminate responders from non-responders, more accurate and much earlier than anatomical imaging. Schulte *et al.*[3] evaluated the use of PET in estimating the grade of tumour regression after neoadjuvant chemotherapy in 27 patients with osteosarcoma prior to surgery. The decrease of FDG uptake showed a close correlation to the amount of tumour necrosis induced by chemotherapy. With a cut-off level of 0.6, all responders and 8 of 10 non-responders could be identified. In an ongoing study in our own hospital in patients with GIST tumours treated with the experimental drug STI 571, a very rapid decline in FDG uptake (as soon as 48hours after the initiation of therapy) was observed in 12/22 patients, often corresponding with symptom relief. In the 6 patients with an early increase in FDG uptake, a rapid deterioration of clinical symptoms was seen. While there was a clear discrimination between responders and non-responders on PET, no major change in tumour volume was observed on CT. With the introduction of new, often non-cytotoxic drugs, no major changes in tumour volume is to be expected and therefor therapy evaluation based on metabolic changes will be become more and more important.

**References**

1 Folpe AL, Lyles RH, Sprouse JT, Conrad EU 3rd, Eary JF. (F-18) fluorodeoxyglucose positron emission tomography as a predictor of pathologic grade and other prognostic variables in bone and soft tissue sarcoma. *Clin Cancer Res* 2000 Apr;6(4)1279–87.
2 Lucas JD, O'Doherty MJ, Wong JC, Bingham JB, McKee PH, Fletcher CD, Smith MA. Evaluation of fluorodeoxyglucose positron emission tomography in the management of soft-tissue sarcomas. *J Bone Joint Surg Br* 1998 May;80(3):441–7.
3 Schulte M, Brecht-Krauss D, Werner M, Hartwig E, Sarkar MR, Keppler P, Kotzerke J, Guhlmann A, Delling G, Reske SN. Evaluation of neoadjuvant therapy response of osteogenic sarcoma using FDG PET. *J Nucl Med* 1999 Oct;40(10):1637–43.

**Local staging of musculoskeletal sarcomas-how good are we?**

A. SAIFUDDIN
*(The Royal Orthopaedic Hospital, Stanmore, Middlesex, United Kingdom)*

Local staging is vital in the planning of surgery for bone and soft-tissue sarcomas. The majority of published work on musculoskeletal tumour staging has dealt with osteosarcoma since this is by far the commonest primary malignant bone tumour. This paper reviews the literature on the local staging of high-grade central appendicular osteosarcoma. It is assumed that the findings can be extrapolated to other bone sarcomas. Imaging techniques available include radiography, scintigraphy, CT and MRI. MRI is currently the technique of choice. The factors that need to be addressed are; 1) intramedullary tumour extent, including identification of 'skip' metastases and relationship of tumour to the growth plate and epiphysis, 2) relationship of tumour to the adjacent joint and 3) relationship to the adjacent major neurovascular structures. Tumour volume is also important in terms of prognosis, particularly for Ewing's sarcoma.

*1) Intramedullary tumour extent:* several studies have assessed the accuracy of MRI by comparing imaging with pathological macroslides. It has been shown that an unenhanced coronal or sagittal T1 weighted spin echo sequence is highly accurate in assessing intramedullary tumour extent. Both STIR and contrast enhanced T1 weighted fat saturated sequences tend to overestimate intraosseous tumour since they are sensitive to adjacent marrow hyperaemia and oedema. MRI can also identify 'skip' metastases, and in this respect is more sensitive than radiography and bone scintigraphy. MR imaging of the whole bone is an absolute requirement. However, no study has been performed that looks at the sensitivity and specificity of MRI in the evaluation of 'skip' lesions. Personal experience indicates that not all additional intramedullary lesions identified at imaging represent 'skip' lesions. Pathological studies have shown that involvement of the growth plate and epiphysis by metaphyseal osteosarcoma occurs in up to 80% of cases. This can be accurately assessed by MRI. Again, an unenhanced T1 weighted sequence is most accurate. Direct evidence of tumour extension is the most reliable finding. The presence of only oedema in the epiphysis is not a specific finding.

*2) Relationship of tumour to the adjacent joint:* intra-articular extension is most commonly a feature of distal femoral osteosarcoma, and typically occurs in the intercondylar notch at the proximal insertion of the anterior cruciate ligament. A pathological fracture into the joint also signifies joint contamination. Both are clearly identified by MRI. However, the important differentiation between intra-capsular/extrasynovial and intra-capsular/intrasynovial extension has not been assessed. The presence of a joint effusion is a common and non-specific finding.

*3) Relationship of tumour to the adjacent neurovascular structures:* this determines the feasibility of limb-salvage surgery and is best assessed using an axial T2 weighted MR sequence. Involvement of the neurovascular structures by extraosseous tumour may be graded on MRI as definitely absent, equivocal or definitely present. In the former and latter cases, MRI correlates accurately with surgical findings. When MRI appearances are equivocal, a safe surgical plane can be achieved in 40–70% of cases. Dynamic enhanced MRI studies can differentiate extraosseous tumour from perineoplastic oedema but the clinical relevance of this needs clarification.

**STS—Which surgical questions need to be answered in future studies?**

R.J. GRIMER
*(Royal Orthopaedic Hospital Oncology Service, Birmingham, UK)*

Surgical aspects of the treatment of STS have largely been ignored in studies of the treatment of STS. Surgical excision of the tumour is however still the largest single intervention that most patients with STS will undergo and thus 'getting it right' is essential. There are many reasons for this including the variety of surgeons who treat them, the large number of centres where they are treated and the near impossibility of doing a randomised controlled trial in any surgical field. However, there are still many surgical questions to be answered and we need to think of ways to approach these. Some of these are:

1. Does it matter who operates on STS?
2. Is there a critical number of sarcomas one should operate on per year to be 'safe'?
3. Is a biopsy essential for retroperitoneal STS?
4. How wide does a wide margin need to be?
5. Is local control important to maximise overall survival?
6. Are there tumours where amputation is the safest option?
7. Is a planned marginal excision safer than an unplanned one?
8. When do you peel vessels of the tumour and when do you reconstruct them?
9. Are two operations better than one?
10. What are the cost / benefit ratios of early plastic surgical reconstruction in 'at risk' sites eg. large adductor compartment tumours?
11. How do you prevent seromas—does it matter?

These are just some of the questions which surgeons ask themselves and their colleagues from time to time. These questions have been circulated in advance of the meeting to an interested group of STS surgeons—their responses have been analyzed and will be discussed in an attempt to define what questions really do need answering and how we might go about this.

**Which questions in radiotherapy need to be answered in future studies?**

B. O'SULLIVAN
*(Princess Margaret Hospital, Toronto, Canada)*

*Introduction:* The use of adjuvant radiotherapy (RT) has greatly expanded the opportunities for conservative surgery in soft tissue sarcoma (STS). Although ample evidence of efficacy exists, the best way to use RT (or sometimes its indications) remains unclear. This presentation will focus on STS and will address a number of continued controversies while recognizing that current strategies provide high rates of local control in many sites.
*Omission of RT:* Undoubtedly a favorable population exists where RT is unnecessary with appropriate surgery (e.g. well contained lesions) but the limits of this approach are uncertain. In contrast, there are unfavourable groups where radiotherapy may also not be useful, and the prognosis is universally poor, e.g. retroperitoneal sarcoma (RPS). Certainly in RPS a randomized trial addressing this issue would be valuable since patients are often destined to die with present approaches, which are both potentially toxic as well as inadequate. In the more favorable lesions criteria for selection are needed and should be applied and studied prospectively to determine selection for surgery alone.
*Reducing Toxicity of RT:* The prospective trials have yielded information but questions remain. In the Canadian SR2 trial, total dose and increased volume was associated with long term limb fibrosis. Strategies to reduce the total dose of radiotherapy delivered, or the biologic dose through exploitation of optimal fractionation approaches have not been addressed prospectively. In the future, it may also be possible to prospectively evaluate radioprotective agents, or replacement of damaged tissue at the macroscopic or cellular level. Opportunities to protect tissues by exclusion from the target volume should be exploited and studied. This is especially important where tissues are critical and dose limiting. The potential to treat RPS with untethered bowel displaced by an unresected tumor offers potential gain with preoperative RT. In this scenario, there is minimal contamination of the abdominal cavity, the tumor vascularity is intact, and the fields are smaller. This approach could readily be the focus of a trial supported by those who are discouraged by the value of post-operative RT in RPS where RT administration is difficult to administer.
*Assessment of Toxicity:* The SR2 trial also revealed that volume irradiated is strongly associated with poor function and limb edema. Yet we are unaware whether this relates to total proportional volume of the limb treated or to proportion of the cross-section of the limb irradiated. In order to address such issues prospective valid and reliable measures of toxicity must continue to be developed and accurate documentation of treatment implemented.
*Definition of Target Volumes:* Because volume is important there is an urgent need to define the volume at risk. Clinico-pathologic studies are needed for radiotherapy planning to permit reduction in normal tissues irradiated. The significance of peritumoral edema on MRI is unknown. This and other imaging knowledge (e.g. PET) must be evaluated to permit optimum surgical and radiotherapy target delineation.
*Optimum Volume and Combined Modality Approaches:* Knowledge of optimum volume will provide the opportunity to combine concurrent chemotherapy with radiotherapy and spare morbidity by treating smaller volumes with conformal plans, high-resolution intensity modulated radiotherapy (IMRT), or brachytherapy. The comparative benefits of these and other approaches (e.g. limb perfusion) requires study from the context of cost, wound healing, function, combined chemoradiotherapy toxicity and, of course, local control.
*Summary:* The evaluations described provide much opportunity for future research in the use of RT in STS and will be discussed with examples.

**Isolated limb perfusion with TNF for locally advanced soft tissues sarcomas: any role outside dedicated centres?**

FERDY J. LEJEUNE
*(Centre Pluridisciplinaire d'Oncologie, CHUV, Lausanne, Switzerland)*

*Background and introduction:* Some sixty percent of Soft Tissue Sarcomas (STS) occur in the limbs Amputation used to be the standard treatment for these extremity lesions but it has now been replaced by limb-sparing surgery in about 90% of patients, without significant impairment of limb function. In the remaining 10% the tumour involves the neurovascular bundle, is very large, is close to an articulation or consists of multiple nodules. In these settings, treatment remains primary amputation and radiotherapy is ineffective. Sadly, however, amputation does not improve life expectancy, because distant micrometastases, mainly pulmonary have usually already grown. We reported that Isolated Limb Perfusion with TNF/IFN gamma and melphalan (TIM-ILP) is an efficient treatment of inoperable soft tissue sarcomas and melanoma of the extremities (1). Moreover, this treatment is a limb-sparing neoadjuvant therapy when it is followed by removal of the tumour remnant(s) (2).
*Method:* The rationale is that isolated perfusion allows high regional concentration of drugs, respectively TNF which targets tumour associated vessels, and melphalan, an alkylating agent, which targets tumour cells (3).
The technique is sophisticated and requires a multidisciplinary team of surgeons with know how in extremity surgical oncology and isolated limb perfusion procedure, anaesthesiologists specialised in intensive care, nuclear medicine specialist for continuous monitoring of leakage to the general circulation, and pump technicians familiarised with hyperthermic isolated limb perfusion. The procedure includes the surgical isolation of the limb major vessels and their connection to the extracorporeal circuit, under tourniquet. For TM-ILP, high dose TNF (3 mg upper or 4 mg lower limb) is administered when no substantial leakage is detected and when tissue temperature reaches 38°C. In the past, for TIM-ILP 0.2 mg IFN gamma was added. High dose melphalan is administered 30 min later, when vascular permeability has been increased by TNF. Perfusion ends after 90 min and the limb vessels are intensively washed of residual drugs. The whole procedure takes around 5 hours. For safety reason, the patient is admitted in intensive care unit for at least half a day.

*Results:* The European TNF/ILP Assessment Group evaluated 260 patients with irresectable STS enrolled in 10 years into four studies with similar protocols to determine whether TM (TNF/melaphalan) or TIM (TNF/IFN gamma/melaphalan)-ILP offers durable limb salvage by effective local control of the tumour with or without subsequent surgery (4, 5). Patients were reviewed by an Independent Review Committee and compared with conventionally treated patients (often by amputation) of a population-based Scandinavian STS Database, since randomised studies were never considered in view of the high response rate and high limb sparing obtained after TM or TIM-ILP.

The proportion of patients who had achieved durable limb salvage ranged from 74% to 87% in the four studies and the objective (complete plus partial) response rates were 56.5–82.6%. The Independent Review Committee considered that 80% of all enrolled patients met the criteria for irresectability. As proven by matched-pair comparison, the ILP treated patients survived as long as conventionally treated patients from the Scandinavian STS Database.

In Lausanne, we reviewed our results as a single centre performing TIM or TM-ILP as a limb salvage treatment for initially non-resectable soft tissue sarcomas of the extremities (6).

Twenty-two patients (6 men and 16 women; 3 upper limb and 19 lower limb tumours) were enrolled. AJCC stage was IIA in 4 patients, III in 7 and IV in 11. Thirteen cases were recurrences or progressions after previous therapy; 5 tumours had a diameter ≥ 20 cm, and 4 were multiple or regionally metastatic. There were 6 malignant fibrous histiocytomas, 5 liposarcomas, 4 malignant schwannomas (malignant peripheral nerve sheet tumours), 3 rhabdomyosarcomas, 2 leiomyosarcomas, 1 recurrent extrasquelettal osteosarcoma and 1 angiosarcoma.

Twenty-four ILPs were performed in the 22 patients; 18 (82%) experienced an objective response: it was complete in 4 (18%) and partial in 14 (64%). Three patients had minimal or no response and the tumour progressed in one case. All patients had fever for 24 hours but only one developed a grade 3 reversible distributive shock syndrome with no sequellae. There was no grade 4 toxicity. Seventeen patients (77%) underwent limb sparing resection of the tumour remnants after a median time of 3.4 months: 10 resections were intracompartmental and 7 extracompartmental. Surgery included flaps or skin grafts in 5 patients, arterial replacement in 2 and knee arthrodesis in 1. Adjuvant chemotherapy was given to 8 patients and radiotherapy to 6. One patient had to be amputated after second ILP.

Secondary amputations were performed for recurrence in two patients, resulting in an overall limb salvage of 19/22 (86%).

After a median follow up of 28 months, 10 recurrences were recorded: 7 were both local and systemic and 3 were only local. The median disease free and overall survival have been > 12.5 and 18.7 months respectively: this is similar to the outcome after primary amputation for similar cases.

*Conclusion:* We conclude that ILP with TNF and chemotherapy is an efficient limb sparing neoadjuvant therapy for *a priori* non-resectable limb soft tissue sarcomas, a condition that only represents 10% of limb STS. The procedure is sophisticated and costly, it requires continuous experience of a multidisciplinary team. For performing at least one ILP per month, only one centre is needed for a 5 million population basin. We recommend the implementation of an European Quality Assurance Programme.

### References

1 Lienard D, Ewalenko P, Delmotte JJ, Renard N and Lejeune FJ 1992, High-dose recombinant tumour necrosis factor alpha in combination with interferon gamma and melphalan in isolation perfusion of the limbs for melanoma and sarcoma: Journal of Clinical Oncology, v. 10, p. 52–60.
2 Eggermont AM, Schraffordt Koops H, Lienard D, Kroon BB, van Geel AN, Hoekstra HJ and Lejeune FJ, 1996, Isolated limb perfusion with high-dose tumour necrosis factor-alpha in combination with interferon-gamma and melphalan for non-resectable extremity soft tissue sarcomas: a multicenter trial [see comments]: Journal of Clinical Oncology, v. 14, p. 2653–65.
3 Lejeune FJ, Ruegg C, and Lienard D, 1998, Clinical applications of TNF-alpha in cancer: Current Opinion in Immunology, v. 10, p. 573–80.
4 Boehringer Ingelheim KG. (Ingelheim, Germany), 1999, Beromun ILP Concept Monograph.
5 Eggermont AM, Gustafson P, Clarke J, Lejeune FJ and Steinmann GG, 2000, A comprehensive efficacy and safety assessment of 260 patients with irresectable soft tissue sarcoma treated from 1988 to 1997 with high dose tumor necrosis factor-alfa and melphalan by isolated limb perfusion. (*submitted*).
6 Lejeune FJ, Pujol N, Lienard D, Mosimann F, Raffaoul W, Genton A, Guillou L, Landry M, Chassot PG, Chiolero R, Bischof-Delaloye A, Leyvraz S, Mirimanoff RO, Bejko D, and Leyvraz PF, 2000, Limb salvage by neoadjuvant isolated perfusion with TNF alpha and melphalan for non-resectable soft tissue sarcoma of the extremities: European Journal of Surgical Oncology, v. 26, p. 669–678.

**Prospective role of chemotherapy combined with hyperthermia in sarcomas?**

ROLF D. ISSELS[1,2] & CLEMENS-M. WENDTNER[1]
*([1]University Hospital Medical Clinic Grosshadern, Medical Clinic III, D-81377 Munich and [2]KKG Hyperthermie, GSF-National Research Center for Environment and Health, D-81377 Munich, Germany)*

*Purpose:* To evaluate the efficacy of neoadjuvant chemotherapy combined with regional hyperthermia (RHT) in adult patients with non-metastatic high-risk soft tissue sarcomas (HR-STS). We report upon the results of two consecutive phase II studies and present a subgroup analysis for patients with retroperitoneal and visceral HR-STS.

*Patients:* 113 patients with HR-STS (non-resectable primary/S1, recurrent/S2, inadequately resected/S3) located within extremities, trunk or abdomen were treated within a neoadjuvant phase II protocol (RHT-91 or RHT-95). HR-criteria were: tumor grade II/III + tumor size (>8cm for RHT-91; >5cm for RHT-95) + extra-compartmental extension. The RHT-91 protocol (59 pts) included 4 cycles of preoperative chemotherapy (XT) plus RHT, followed by surgery and 4 cycles of adjuvant XT plus RHT. In addition, R1/R2-resected pts received radiation. The RHT-95 protocol (54 pts) was identical except that pts after surgery obtained XT without RHT and adequate radiation regardless of resection status. XT of both studies consisted of etoposide (125mg/m$^2$) on day 1+4, ifosfamide (1500mg/m$^2$) on day 1 to 4, and adriamycin (50mg/m$^2$) on day 1 (EIA). RHT (1hr at 42.5°C) was given on day 1+4.

*Results:* Radiographic response in 52 evaluable pts of the RHT-91 (42%) and in 32 assessable pts of the RHT-95 study (28%) included 1+0 CR, 8+5 PR, 13+4 MR, 17+10 SD and 13+13 PD, respectively. Among 74 pts undergoing surgery, amputation rate was <15%. After different median observation times (RHT-91: 58mo/ RHT-95: 30mo) probability of overall survival (42% vs 48%; p = 0.392) and distant progression free survival (51% vs 64%; p = 0.357) are quite similar for both studies. Subgroup analysis (S3 vs S1, S2) for overall survival revealed also no significant difference (47% vs 48%; p = 0.616). Interestingly, local relapse free survival was in favour of the RHT-91 study, which included pre- and postoperative RHT (58% vs 57%; p = 0.021).

Among these patients, we performed also a subgroup analysis for 58 HR-STS with retroperitoneal or visceral (RP/V) STS. Radiographic response after neoadjuvant thermochemotherapy was assessable in 40 patients (5 PR, 8 MR) with no overall response rate of 33%. After completion of protocol treatment, 25 patients (43%) had no evidence of disease (NED). 5-year probabilities of only local failure-free, any local failure-free, distant metastasis-free, event-free and overall survival (OS) were 53%, 25%, 51%, 20% and 32%, respectively. Median OS was 31 months for the entire study cohort, while OS was in favor of patients with NED vs. NON-NED status (76 vs. 20 months; $P = .0001$). Patients responding to neoadjuvant thermochemotherapy associated significantly with NED ($P = .008$). At 5-year follow-up, probability of OS was in favor of patients responding to neoadjuvant thermochemotherapy (60% vs. 10%; $P = .0014$).
*Conclusions:* Based on these results, a randomized prospective phase III intergroup study (EORTC 62961/ESHO RHT-95) with transatlantic participation is ongoing comparing EIA +/- RHT in previously defined (S1-S3) risk groups and includes pre- and post-operative RHT in the experimental treatment arm. Since July 1997 more than 100 pts have been randomized and treatment within this protocol is feasible and safe while impact on local disease control and survival has to be awaited.
Supported by a grant of Deutsche Krebshilfe

**Why have these new local treatments not become more widespread? General discussion**

M.H. ROBINSON
*(Cancer Research Centre, Weston Park Hospital, Sheffield, UK)*

There have been up to 80 reports of the use of isolated limb perfusion in the treatment of extremity soft tissue sarcoma since its introduction in 1985. It is a technique whose use is almost entirely confined to parts of Europe and which has not gained widespread acceptance. However, the multitude of published studies have clearly indicated that perfusion techniques are capable of producing complete remission rates of up to 30% with 80% of limbs being spared amputation. These remission rates cannot be achieved by other more widely used techniques such as pre-operative radiotherapy with or without chemotherapy. The issue is therefore why hasn't ILP gained wider acceptance? The answers revolve around the indications, results, complications and alternatives for these novel techniques.
The first issue is patient selection. One surgeon's selection criteria for amputation may be different to another. In a busy sarcoma unit only 5% of patients may require an amputation as treatment for their extremity sarcoma. This means that only a small proportion of referred patients are suitable for experimental local techniques. Other options are less technically demanding e.g. pre-operative chemo-radiotherapy. The superiority of HILP over these techniques has never been convincingly proven. ILP is a complex technique, which has a considerable potential for side effects, which requires considerable surgical experience for its safe delivery. TNF alpha is severely toxic if it escapes into the systemic circulation. There is debate in the literature around the other potential side effects. Klicks (1998)[1] found only 10 vascular complications in 466 ILPs (2.1%), all occurring in women. Drory (1998)[2] reported minimal clinical signs of peripheral nerve damage in 7/10 patients undergoing HILP. This was mild, didn't disturb function and improved over time. The safety of radiotherapy with HILP is also debated. Vrouenraets (1997)[3] reported considerable risk of tissue necrosis and impaired healing when they were used together. Olieman (1998)[4] found no difference in morbidity.
How do practitioners of novel techniques such as HILP and other hyperthermic procedures convince the rest of us that investment in the time and money required to safely deliver them is worthwhile? Not easily is the answer. One more difficulty in the way is the uncertain connection between local control and improvements in survival. Patients selected for these procedures usually have a very high risk (>50%) of developing fatal metastases even where local control is achieved. A recent European Consensus meeting suggested that proponents of these techniques should carry out a prospective study in which the patients are selected for treatment by an independent panel of experts. The alternative is a randomized trial, which seems impractical. A detailed study of consequent limb function would be essential as treatment is palliative in a significant proportion.
The evaluation of new techniques is a challenge to modern money conscious health care systems. In Britain new treatments (especially drugs) are subjected to thorough examination by the National Institute of Clinical Excellence before being made available on the NHS. Such a review of the value of HILP would undoubtedly return a verdict of 'not proven'.

**References**

1 Klicks, RJ, *et al.* (1998). Vascular complications of isolated limb perfusion. *Eur J Surg Oncol*, 24, 288–91.
2 Drory, VE, *et al.* (1998). Neurotoxicity of isolated limb perfusion with tumor necrosis factor. *J Neurol Sci*, 158, 1–4.
3 Vrouenraets, BC, *et al.* (1997). Complications of combined radiotherapy and isolated limb perfusion with tumor necrosis factor alpha +/– interferon gamma and melphalan in patients with irresectable soft tissue tumors. *J Surg Oncol*, 65, 88–94.
4 Olieman, AF, *et al.*, (1998) Feasibility and efficacy of external beam radiotherapy after hyperthermic isolated limb perfusion with TNF-alpha and melphalan for limb-saving treatment in locally advanced extremity soft-tissue sarcoma. *Int J Radiat Oncol Biol Phys*, 40, 807–14.

**Adjuvant chemotherapy has no effect on survival—the EORTC experience.**

V.H.C. BRAMWELL
*(London Regional Cancer Centre, 790 Commissioners Road, East, London, Ontario, Canada N6A 4L6)*

*Purpose:* In the past 25 yrs, the EORTC STBSG has conducted 3 randomized trials (RCT) evaluating the role of adjuvant chemotherapy in adult patients with soft tissue sarcomas (STS). Presented here are updated results for trial 62771 (Bramwell et al. J Clin Oncol 12:1137, 1994), and a recent analysis of trial 62874 (Gortzak et al. Eur J Cancer, 37:1096–1103, 2001). Trial 62931 is in progress.

| Trial #/dates | Patients entered | Eligible | Patient characteristics | Chemotherapy (CT)* |
|---|---|---|---|---|
| 62771<br>1977–1988 | 468 | 317 | age 15–70<br>all sites, grades | CYVADIC × 8<br>post-surgery |
| 62874<br>1988–1995 | 150 | 134 | age 15–75<br>high risk (grade II, III or size >8cm<br>or recurrent/residual) all sites | DOX (50 mg/m$^2$)<br>IFOS (5g/m$^2$)<br>neoadjuvant |
| 62931<br>1995– | ongoing<br>target accrual | <br>340 | age 16–70<br>grade II, III all sites | DOX (75 mg/m$^2$)<br>+ IFOS (5 g/m$^2$)<br>+ G-CSF post-surgery |

*All patients receive optimal local treatment (complete resection ± radiotherapy) and are randomized between control and chemotherapy arms DOX = Doxorubicin; IFOS = Ifosfamide; CYVADIC = Cyclophosphamide//vincristine/DOX/Dacarbazine

| Trial #/arm | Eligible patients | Follow-up (median) | 5 yr% | | | 10 yr% | | |
|---|---|---|---|---|---|---|---|---|
| | | | LR | RFS | OS | LR | RFS | OS |
| 62771 control | 172 | >120 mos | 30 | 46 | 62 | 30 | 45 | 54 |
| CT | 145 | | 16 | 60 | 64 | 19 | 57 | 54 |
| 62874 control | 67 | 88 mos | 12 | 52 | 64 | | | |
| CT | 67 | | 12 | 56 | 65 | | | |

*Patients & methods:* Trial accrual and design are summarized below:
*Results:* For trial 62771, there is no significant difference (p = 0.84) in overall survival (OS), despite significant reductions (p = 0.008 and 0.006 respectively) in local recurrence (LR) and relapse free survival (RFS) favoring CYVADIC. A reduction in LR is only apparent in the group of head, neck and trunk sarcomas (p = 0.002) but not in limb sarcomas (p = 0.31).
For trial 62874, there also are no significant differences in RFS (p = 0.35) and OS (p = 0.22), nor in the incidence of LR.
*Discussion:* Despite trial 62771 being the largest RCT to date of adjutant CT in STS, it was not possible to demonstrate a significant improvement in OS for CT, a result reflected in the SMAC meta-analysis (Lancet 350:1647, 1997) to which it contributed ≏30% of patients. Strengths and limitations of this study will be critically reviewed. However, reduced LR rates were felt to indicate a positive effect of CT and this was explored further in trial 62874. Designed as a phase II feasibility study of neoadjuvant CT, this latter trial was underpowered to detect a significant difference in 5 yr OS. Toxicity was tolerable and chemotherapy did not interfere with planned surgery, nor affect postoperative wound healing. However, slow accrual highlighted difficulties in recruitment to a multicentre neoadjuvant protocol. Although inter-trial comparisons are subject to bias, in trial 62874 the lower rate of local recurrence in high risk STS is encouraging, and perhaps reflects advances in local management. Dose intensification of DOX in trial 62931 remains a relevant question, but accrual to this study is slow.

**Italian experience on adjuvant chemotherapy of adult soft tissue sarcomas.**

S. FRUSTACI*, A. DE PAOLI*, A. BUONADONNA* & P. PICCI°.
*(Italian Sarcoma Group (ISG). *C.R.O., Aviano, °Rizzoli, Bologna; Italy)*

The role of CT in STS represents a matter of debate. No conclusive data have been obtained from the concluded trials. However, some points deserve discussion. *FIRST GENERATION TRIALS:* From 1973 to 1990, 14 randomized trials have been conducted. The intrinsic bias was: broad selection criteria; use of inactive agents (VCR, CTX), low doses of the actives ones; low number of accrued patients. *META-ANALYSIS:* The first, performed on individual patients records, revealed an impact on local-DFS, distant-DFS but only a trend for the OS. In the subgroup of extremities including 886 pts, also the OS was statistically different between CT and control (FU)(p = 0.029). *SECOND GENERATION TRIALS*: In the early '90s, further prospective randomized trials were designed worldwide and in some instances activated by different groups. Their main differences were: restricted selection criteria (high risk), use of antracyclines and ifosfamide only, hematopoietic growth factors and the limited number of cycles. The differences are: dose-intensity, time of starting chemotherapy. RESULTS OF THE ISG TRIAL:
*—intention to treat-analysis:* From 6/92 to 11/96, 104 pts were assigned to CT or FU. The interim analysis was presented(ASCO '97; Abs.N°c *1785) and the further yearly analyses were also reported(ASCO'99;ABS.N°c*2108). The mature results, obtained after a median follow-up of 59 months still confirm the advantage of the chemotherapy group in comparison with the control group in terms of survival and overall disease free survival. The major advantage is however obtained in delaying the relapse either locally or at the lung level, whereas the amount of distant events is similar in both arms (JCO, vol. 19(5);2001).
*—subgroups analysis:* Beside the intention to treat analysis, further aspects have been looked at: *Efficacy of chemotherapy* (efficacy), exclusion from the analysis of those patients never starting chemotherapy (7 patients in the treatment group); *Impact of the dose intensity* (D.I.), comparison of those pts receiving a D.I. ≥85% versus <85% and versus the control group; *Evaluation of the post-relapse survival* (Post-rel-Surv), comparison of the survival of treated and untreated groups after the relapse. *Preliminary data*: the analysis of the three above mentioned aspects also seem to indicate the usefulness of adequate chemotherapy in the adjuvant setting in a high risk patient population.
*Conclusions:* The present Italian trial, with an adequate follow-up, strongly indicates the activity of adjuvant chemotherapy in a high-risk patient population. This efficacy and gain in survival is however mainly due to a delay in relapse either local or at distant sites rather than a real decrease in distant events. However, this substantial delay is worthwhile in young patients and determined the statistically difference between the untreated and treated groups. In Italy, the participating Institutions regularly offer adjuvant chemotherapy to patients presenting with the same high-risk characteristics.

**Pro adjuvant treatment outside clinical trial: Adjuvant chemotherapy is indicated for stage III soft-tissue sarcomas of the extremities**

R.S. BENJAMIN
*(UT MD Anderson, Houston, USA)*

The role of Adjuvant Chemotherapy in the treatment of patients with soft-tissue sarcomas remains controversial despite a number of randomized trials and several meta-analyses. Of the several, mostly small, randomized trials of doxorubicin-based chemotherapy with varying eligibility criteria regarding size, grade, and primary tumor site, only one showed survival benefit. The NCI study was the first to indicate that extremity tumors might benefit to a greater extent than those of other primary sites. Only one study showed better disease-free survival in the observation group than in the chemotherapy group, whereas 8/9 studies showed better disease-free survival. Moreover, while none of the studies showed improved overall survival for the control group (one showed equal survival), 8/9 showed improved survival in the adjuvant chemotherapy group.
Zalupsky and Baker were the first to perform such a meta-analysis using the published data on 926 patients with extremity sarcomas included in these studies. The meta-analysis demonstrated statistically significant improvement in both disease-free survival and overall survival. The absolute improvement in disease-free survival at 10 years was 15% ($p<.0001$), and absolute improvement in survival at 10 years was 10% ($p=.0005$). This study was criticized severely because it used published data rather than raw data.
Formal results of a meta-analysis of individual patient data showed statistically significant improvement in local-relapse-free survival, distant-metastasis-free survival, and overall disease-free survival with a trend ($p = 0.12$) for improved survival. For the 886 patients with extremity soft-tissue sarcomas, there was statistically significant improvement in distant-metastasis-free survival, in overall disease-free survival, and overall survival. The risk of relapse (local and distant) decreased by 32% (absolute improvement at 10 years, 15%), the risk of distant metastasis decreased by 34% (absolute improvement at 10 years, 13%), and the risk of death decreased by 20% (absolute improvement at 10 years, 8%). The magnitude of improvement is equivalent to that of 5-FU and leucovorin in colon cancer or CMF in breast cancer.

There is only one modern study of adjuvant chemotherapy reported by the Italian Cooperative Group in the March, 2001 issue of the JCO. That study, discussed in further detail at this meeting by Dr. Frustaci, demonstrated a 19% overall survival benefit at 4 years for the patients treated with adjuvant chemotherapy, and it is the only study to target the appropriate group of patients for an adjuvant chemotherapy study: those with deep, high-grade soft-tissue sarcomas of the extremities >5 cm in primary size, *i.e.* current stage III. No one has ever claimed that adjuvant chemotherapy produces substantial survival benefit for patients with soft-tissue sarcomas. Rather, as with other solid tumors, benefits are small but real, and the chemotherapy for soft-tissue sarcomas is highly toxic, and dose-intensive therapy is required for optimum efficacy. The neoadjuvant strategy, successful in osteosarcoma and other tumors, allows selection of patients who will benefit most from chemotherapy and is considered standard treatment for stage III tumors at our institution.

**Adjuvant therapy for soft tissue sarcoma**

K.H. ANTMAN
*(Division of Medical Oncology, Department of Medicine, Columbia University, New York, USA)*

Adjuvant chemotherapy is currently established in the treatment of rhabdomyosarcomas, osteosarcomas, and Ewing's sarcomas, but remains unproven in other adult soft tissue sarcomas and therefore should be used only in the context of a properly conducted clinical trial. Given the significantly improved local control in the metaanalysis, however, preoperative chemotherapy may be part of a multimodality treatment plan for borderline resectable soft tissue sarcomas. The risks of adjuvant chemotherapy trials are not currently warranted in patients with low-grade lesions, given their low probability of metastatic spread. Because surgical salvage of pulmonary metastases or recurrent local disease is possible in some patients, disease-free survival may be a less meaningful endpoint than overall survival.

Of the 12 reported adjuvant studies, only 2 (one from the Rizzoli Center in Bologna[1] and the other from the Foundation Bergonie in Bordeaux) show a significant overall survival advantage for chemotherapy.(table 1). One study (European Organization for Research in the Treatment of Cancer (EORTC)) demonstrated a significant improvement in local control for adjuvant chemotherapy, but no overall survival benefit.[2] Subset analyses in two additional studies (from M.D. Anderson Hospital[3] and the National Cancer Institute[4]) currently indicate a significant disease-free survival advantage for adjuvant chemotherapy in extremity lesions but no significant improvement in survival. (While the NCI reported a significantly prolonged survival for the subset of chemotherapy-treated extremity primaries on initial reports,[5] survival is no longer significantly different, due to late relapses.[4] In the subset analysis of the same NCI study for retroperitoneal sarcomas, the survival of the control group is superior to the treatment group. Of the 12 randomized trials, the survival of the observation arm exceeds that of the chemotherapy arm in 3 studies (Mayo Clinic, Eastern Cooperative Oncology Group (ECOG), Scandinavian study). Doxorubicin-associated cardiotoxicity has occurred in about 10% of treated patients, occasionally contributing to treatment-related deaths. Based on these data, adjuvant chemotherapy should be considered investigational for adult soft tissue sarcomas of any primary site.

**Table 1.** *Randomized adjuvant trials in high grade soft tissue sarcomas*

| | | %DFS | | | %S | | | |
|---|---|---|---|---|---|---|---|---|
| Institute | Drugs | N | - | + | p | - | + | p |
| EORTC[2] ACVD | 468 | 43 | 56 | .007 | 56 | 63 | NS | |
| **Extremities** | | 233 | 64 | 64 | NS | 55 | 42 | NS |
| Bordeaux[7] | ACVD | 59 | 16 | 57 | <.01* | 53 | 87 | <.01 |
| **Extremities** | | 36 | NA | NA | NA | NA | NA | NA |
| Mayo Clinic[8] | AVDAd | 61 | 68 | 65 | NS | 70 | 70 | NS |
| **Extremities** | | 48 | 67 | 88 | .08 | 83 | 63 | NS |
| MD Anderson[3] | ACVAd | 47 | 83 | 76 | NS | NA | NA | NS |
| **Extremities** | | 43 | 35 | 54 | <.05 | 46 | 65 | NS |
| NCI[4,5,9,10] | ACM | | | | | | | |
| Trunk | | 22 | 47 | 92 | <.01 | 61 | 82 | NS |
| Retroperitoneal | | 15 | NA | NA | NA | 100 | 47 | .06# |
| other | | 31 | 49 | 77 | .075 | 58 | 68 | NS |
| **Extremities** | | 67 | 28 | 54 | <.05 | 60 | 54 | NS |
| Scandinavia[11] | A | 181 | 56 | 62 | NS | 70 | 75 | NS |
| **Extremities** | | 155 | NS | NS | NS | NS | NS | NS |
| Pooled DFCI/MGH, ISSG, ECOG[12] | A | 168 | 53 | 66 | NS | 65 | 68 | NS |
| **Extremities** | | 72 | 64 | 79 | NS | 70 | 79 | NS |
| GOG[13] | A | 156 | 47 | 59 | NS | 52 | 60 | NS |
| UCLA[14] **Extremities** | A | 119 | 54 | 56 | NS | 74 | 78 | NS |
| Rizzoli[1,15] **Extremities** | A | 77 | 45 | 73 | <.05 | 70 | 91 | <.05 |

Abbreviations: DFS, disease-free survival; S, survival; -, without chemotherapy; +, with chemotherapy; EORTC, European Organization for the Research and Treatment of Cancer; DFCI/MGH, Dana Farber Cancer Institute/Massachusetts General Hospital; ISSG, Intergroup Sarcoma Study Group; ECOG, Eastern Cooperative Oncology Group; GOG, Gynecologic Oncology Group; UCLA, University of California at Los Angeles; NCI, National Cancer Institute; A, doxorubicin (doxorubicin); V, vincristine; Ad, Actinomycin D; C, cyclophosphamide; D, dacarbazine; NA, not available; NS, not significant

*Authors reported metastatic-free survival.

### 2 year survival inferior in chemotherapy arm.

A meta-analysis of individual patient data from all randomized adjuvant trials in sarcoma included data from 1568 patients. Adjuvant chemotherapy improved the time to local and distant recurrence-free survival (hazard ratios 0.73 and 0.70 with p-values of 0.016 and 0.0003, respectively). However, for overall survival, the absolute difference was a nonsignificant 4% advantage for chemotherapy and a hazard ratio of 0.89 (p = 0.12).[6]

Randomized trials thus far of ifosfamide and doxorubicin based regimens are small with short follow-up, but will be of interest.

**References**

1 Picci P, Bacci G, Gherlinzoni F, *et al.* Results of a randomized trial for the treatment of localized soft tissue tumors of the extremities in adult patients. In: Ryan JR, Baker LO, eds. Recent concepts in sarcoma treatment. Dordrecht, Netherlands: Kluwer Academic Publishers, 1988:144–148.
2 Bramwell V, Rouesse J, Steward W, *et al.* Adjuvant CYVADIC chemotherapy for adult soft tissue sarcoma: reduced local recurrence but no improvement in survival. J Clin Oncol 1994; 12:1137–1149.
3 Lindberg RD, Murphy WK, Benjamin RS, *et al.* Adjuvant chemotherapy in the treatment of primary soft tissue sarcomas: preliminary report. Management of Bone and Soft Tissue Tumors. Chicago: Year Book Medical Publishers, Inc, 1977:343–352.
4 Baker AR, Chang AE, Glatstein E, Rosenberg SA. National Cancer Institute experience in the management of high-grade extremity soft tissue sarcomas. In: JR R, LO B, eds. Recent concepts in sarcoma treatment. Dordrecht, Netherlands: Kluwer Academic Publishers, 1988:123–130.
5 Rosenberg SA, Tepper J, Glatstein E, *et al.* Prospective randomized evaluation of adjuvant chemotherapy in adults with soft tissue sarcomas of the extremities. Cancer 1983; 52:424–434.
6 Collaboration SM-a. Adjuvant chemotherapy for localized resectable soft-tissue sarcoma of adults: meta-analysis of individual data. Lancet 1997; 350:1647–54.
7 Ravaud A, Nguyen BB, Coindre JM, *et al.* Adjuvant chemotherapy with CyVADIC in high-risk soft tissue sarcoma: a randomized prospective trial. In: Salmon SE, ed. Adjuvant Therapy of Cancer VI. Philadelphia: W B Saunders Company, 1990:556–566.
8 Edmonson JH, Fleming TR, Ivins JC, *et al.* Randomized study of systemic chemotherapy following complete excision of nonosseus sarcomas. J Clin Oncol 1984; 2:1390–1396.
9 Glenn J, Sindelar WF, Kinsella T. Results of multimodality therapy of resectable soft-tissue sarcomas of the retroperitoneum. Surgery 1985; 97:316–325.
10 Glenn J, Kinsella T, Glatstein E, *et al.* A randomized, prospective trial of adjuvant chemotherapy in adults with soft tissue sarcomas of the head and neck, breast, and trunk. Cancer 1985; 55:1206–1214.
11 Alvegard TA, Sigurdsson H, Mouridsen H, *et al.* Adjuvant chemotherapy with doxorubicinin high-grade soft tissue sarcoma: A randomized trial of the Scandinavian Sarcoma Group. J Clin Oncol 1989; 7:1504–13.
12 Antman K, Ryan L, Borden E, *et al.* Pooled results from three randomized adjuvant studies of doxorubicin versus observation in soft tissue sarcoma: 10-year results and review of the literature. In: Salmon S, ed. Adjuvant Therapy of Cancer VI. Philadelphia: WB Saunders Company, 1990:529–544.
13 Omura GA, Major FJ, Blessing JA, *et al.* A randomized clinical trial of adjuvant Adriamycin in uterine sarcomas: a Gynecologic Oncology Group study. J Clin Oncol 1985; 3:1240–1245.
14 Eilber FR, Giuliano AE, Huth JF, Morton DL. Adjuvant Adriamycin in high-grade extremity soft-tissue sarcomas—a randomized prospective trial. Proc Am Soc Clin Oncol 1986; 5:125 (abstract C-488).
15 Gherlinzoni F, Bacci G, Picci P, *et al.* A randomized trial for the treatment of high-grade soft-tissue sarcomas of the extremities: preliminary observations. J Clin Oncol 1986; 4:552–558.

**Is Doxorubicin really the standard treatment of advanced soft tissue sarcomas?**

T. ALVEGAARD

*(Southern Swedish Regional Tumour Registry, University Hospital, Lund, Sweden)*

*Introduction:* Adult soft tissue sarcoma (STS) is considered to be moderately sensitive to chemotherapy, with some reports suggesting that tumours of high-grade malignancy respond better than low-grade tumours. Doxorubicin and ifosfamide are generally considered to be the most active agents, and aggressive combination regimens have yielded response rates of around 45%. However, the role of chemotherapy in prolonging survival is still undefined in advanced disease and there is a need for the development of new chemotherapy approaches. Etoposide is considered to have low activity in adult STS. However, a recent study suggested that the activity of etoposide is considerably higher when administered as a prolonged infusion, taking advantage of the high cell cycle phase specificity of this agent. Combined with a moderate ifosfamide dose (4500 mg/m2), etoposide 600 mg/m2 given as a 72 h infusion yielded a 40% overall response rate in high-grade adult STS. This finding prompted the Scandinavian Sarcoma Group (SSG) to perform a prospective phase II trial to establish the level of activity for this regimen in adult STS. The study also addressed the possibility of dose escalation with the addition of granulocyte colony-stimulating factor (G-CSF) (VIG regimen), and analysed the impact of G-CSF on haematological toxicity. Finally, in selected patients, the study analysed disease-free and overall survival after chemotherapy and surgical removal of all identifiable disease.

*Patients and methods:* The SSG 10/91 study was open to patients aged 15–70 years, with WHO performance status 0–2, histologically proven soft tissue sarcoma and measurable locally advanced or metastatic STS. Before chemotherapy, tumour measurements were taken by CT of MRI (magnetic resonance image) scans. Tumours of all malignancy grades were eligible, but small-cell STS (extraskeletal Ewing's sarcoma, primitive neuroectodermal tumours and undifferentiated variants) were ineligible, as were patients treated with radiotherapy to the indicator lesion(s) during the preceding 2 months. Other requirements were adequate renal function (creatinine clearance >70ml/min), WBC count >3.0 × 109/1 and platelet count >100 × 109/1.

Of 92 eligible patients (median age 51 years), 85% had tumours of high-grade malignancy and 82% had metastatic disease. Chemotherapy, the baseline dose, consisted of etoposide 600 mg/m2 as a 72 h infusion and ifosfamide 1500 mg/m2/day for 3 days, followed by G-CSF support. Stepwise 10% dose escalations were performed depending on haematological toxicity. For patients considered operable after induction chemotherapy, surgical resection of all identifiable residual tumours was attempted.

*Results:* Complete and partial response rates were 11% and 31%, for an overall response rate of 42% (95% CI 31–52%). Forty-eight per cent of courses were dose escalated by a median of 20%. Complete responders had significantly higher, and patients with progressive disease had significantly lower, dose levels than other patients. None of the 20 patients with liver metastases responded despite high dose levels. The addition of G-CSF led to significantly higher dose levels, improved schedule adherence and less haematological toxicity, but no apparent increase in response rate.

*Discussion:* The present study demonstrated a high overall response rate (42%) for the present etoposide/ifosfamide combination. The present response rate is similar to that reported for the most potent ifosfamide/doxorubicin combinations. However, there is still controversy regarding whether combination chemotherapy is in fact superior to single-agent doxorubicin in adult STS. In randomised studies, response rates are commonly lower than in preceding phase II studies, as demonstrated in a recent randomised EORTC study where no difference could be found between doxorubicin alone, a doxorubicin/ifosfamide combination and CYVADIC. Considering the low level of non-haematological toxicity observed in this study, the VIG-regimen may be an attractive alternative to doxorubicin-containing chemotherapy. The study supports the concept that surgical removal of metastatic disease may prolong survival and that some patients may be cured by metastasectomy. An escalated dose of etoposide adm! inistered as a prolonged infusion may increase this agent's activity against STS, taking advantage of phase specificity. The VIG regimen is well tolerated, and the addition of G-CSF appears to reduce haematological toxicity and the incidence of neutropenic fever, to allow dose escalation and to improve schedule adherence.

**New drugs for treatment of metastatic soft tissue sarcomas?**

A. LE CESNE
*(Institut Gustave Roussy, Villejuif, France)*

Results of first-line chemotherapy in adult advanced soft-tissue sarcoma remain disappointing. Only two drugs, doxorubicin and ifosfamide, have demonstrated a consistent single-agent activity yielding response rates of 10 to 25%. Dacarbazine seems to have only a minor activity, if any, in leiomyosarcomas. If there is some evidence of a dose-response relationship for doxorubicin and ifosfamide in soft-tissue sarcoma, the objective response rate reported with doxorubicin alone have progressively decreased during the last 10 years in trials coordinated by cooperative groups. In addition, despite higher response rates achieved in some studies, no multidrug regimen has demonstrated any advantage in terms of overall survival, when compared to single agent Doxorubicin, given at optimal doses. In the field of sarcoma new active drugs are urgently needed. One of the major goals in clinical trials assessing activity of new agents in these well-known advanced chemoresistant tumours is to identify on a little cohort of patients potential active drugs to definitive inactive agents. Due to the large heterogeneity of these tumours and patients included in phase II studies, the results are often inconclusive and controversial. Different histological subtype behave like different diseases with different chemosensitivity (liposarcoma vs. leiomyosarcoma) Therefore, response to chemotherapy (complete and partial response) and overall survival are not predicted by the same factors in retrospective analysis; in consequence of which, response should not be the only end-point for evaluation of new agents and combinations in this disease.

In terms of objective response (WHO criteria of response), which defines today the activity of chemotherapy or biotherapy, the results observed with new drugs are disappointing. None of these following agents: taxanes, liposomal doxorubicin, gemcitabine, topotecan, ET-743 gives more than 15% of objective response in palliative situations. However, if we carefully analysed these studies, a relevant activity of these drugs could be find in some selected histological subtype of sarcomas: gemcitabine in uterine leiomyosarcomas, ET-743 in leiomyosarcomas, paclitaxel in angiosarcoma. The era of selected drugs for selected sarcomas is open with these new drugs and by the emergence of new concepts: specific agent for specific tumour targets. The dramatic activity of STI-571 on GIST (histological subtype uniformly resistant to doxorubicin and/or ifosfamide regimens) and the interesting mechanism of action of rosiglitazone in dedifferenciated abdominal liposarcoma have to modify our strategic approaches in the management of patients with advanced sarcomas.

In a context of palliative therapeutic approaches, we have to favour a prolonged stable disease achieved without major toxicity over transient partial responses achieved at the cost of life-threatening side effects. The number of patients experiencing prolonged stabilisation of their disease in phase II and III trials can modify the progression free survival of patients included in these trials and this clinical parameter have to take into consideration for evaluation of activity of new drugs. The WHO criteria of response could integrate in the next future the tumour status at the end of the planned chemotherapy schedule and the median duration of the disease stabilisation in metastatic soft tissue sarcoma, since a high proportion (30 to 60%) of patients with metastatic soft tissue sarcomas experiences this type of response with tumour changes insufficient to enable classification into distinct response categories (CR, PR and PD).

With the emergence of inhibitors of angiogenesis, new non cytotoxic agents and new cellular targets for gene therapy programs and cancer vaccines, one of the objectives of the next century will undoubtedly be to significantly prolong life of these patients, by maintaining a 'dormancy-state' in non symptomatic lesions. On the other hand, dose-intensified combinations, still as part of study protocols, had to be always proposed in highly selected patients with highly chemosensitive-advanced sarcomas.

In addition, the well-known discrepancy between clinical and histological response in locally advanced soft tissue sarcoma treated with preoperative chemotherapy can be easily applied to metastatic sarcomas. Histological responses could be a more accurate predictor of chemosensitivity than clinical responses, making difficult to evaluate the actual impact of these new drugs on disease free and overall survival of these patients.

**The experience of SFOP in the treatment of Ewing/PNET family of tumours**

O. OBERLIN
*(Institut Gustave Roussy, Villejuif, France)*

The purposes of the SFOP EW88 study were 1) to improve survival rates in patients with Ewing's sarcoma (ES) or peripheral neuroectodermal tumours (PNET) using semi-continuous chemotherapy and performing resection of the primary, as often as possible, 2) To identify prognostic factors. 141 patients with localised tumour were entered onto the trial between January 1988 and December 1992. Induction therapy consisted of 5 courses of Cytoxan, 150 mg/m2 × 7 days, followed by Doxorubicin, 35 mg/m2 IV on day 8. Surgery was recommended whenever possible. The delivery of radiation therapy was based on the quality of resection and the histological response to CT. Maintenance chemotherapy consisted of vincristine + actinomycin and cytoxan + doxorubicin.

*Results:* After a median follow-up of 8.5 years, the projected overall survival at 5 years was 66% and disease-free survival (DFS) was 58%. In patients treated by surgery, only the histological response to CT had an influence on survival: 75% DFS for patients with a good histological response (less than 5% of cells), 48% for intermediate responders and only 20% for poor responders (> 30% of cells), $p < 0.0001$. The tumour volume by itself had no influence on DFS in these patients. In contrast, the tumour volume had a strong impact on DFS in patients treated by radiation therapy alone. Age had no impact on outcome. As size of the primary and response to chemotherapy are independently correlated with outcome. We could identify 3 risk groups on the base of these factors:

- The standard risk group included the tumours with good response to chemotherapy (less than 5% viable cells) and the small unresected tumours but who had good clinical response. Its disease-free survival was 75%.
- The high risk group included the tumours with clinically or histologically poor response to chemotherapy (with more than 30% viable cells). Its disease-free survival was only 13%.
- The intermediate risk group included the tumours with intermediate response to chemotherapy (between 5 to 30% viable cells) and the large unresected tumours but which had a good clinical response. It's disease-free survival was 44%.

The subsequent SFOP EW93 study was based on this risk groups. In the intermediate risk group, the role of the adjunction of VP16 + ifosfamide to the previous chemotherapy was evaluated. In the high risk group, the value of high dose chemotherapy with stem cell support was addressed. The preliminary results of this study will be presented.

**Lessons from the EICESS 92 study**

H. JÜRGENS, B. FRÖHLICH, S. AHRENS, M. PAULUSSEN, A. ZOUBEK, I. LEWIS, P.A. VOÛTE, W. WINKELMANN, B. DOCKHORN–DWORNICZAK & A.W. CRAFT
*(Department of Paediatric Haematology and Oncology, University of Münster, Germany)*

The EICESS 92 protocol for the treatment of patients (pts) with localised and primary metastatic Ewing tumours (ET) was initiated in 1992 as a cooperative project of the German Society of Paediatric Oncology and Haematology with its associated partners in Austria, the Netherlands, and Switzerland, and the United Kingdom Children's Cancer Study Group. Between 04/92 and 12/99, 643 ET pts with a median age of 15 (0,8–35) years and a female-to-male ratio of 40% females were registered. The primary tumour was mostly localised in the pelvis (25%) and the femur (19%). According to tumour volume (TV) and the presence of primary metastases as established risk factors, the pts were allocated into two risk strata. The standard risk (SR) group included 154 pts with localised ET and a TV <100 ml, the high risk (HR) group, 489 pts with a TV >100 ml and/or primary metastatic disease. SR pts were randomised for maintenance chemotherapy to receive the standard VAIA (vincristine, adriamycine, ifosfamide, actinomycin D) or the less intensive VACA regimen (vincristine, adriamycine, cyclophosphamide, actinomycin d), and HR pts were randomised for standard VAIA versus more intense EVAIA (VAIA plus additional etoposide) chemotherapy.

After a median time under study of 52 months, overall survival (OAS) and event free survival (EFS) five years after diagnosis were 0.69 and 0.59 in pts with localised ET and 0.42 and 0.32 in pts with primary metastases. In pts with localised ET the analysis according to risk groups shows a significantly better five-year EFS in SR pts (0.67) compared to HR pts (0.55) ($p = 0.0018$). There was no significant difference in outcome between VAIA and VACA in the SR group and between VAIA and EVAIA in the HR group, but life table analyses show in both groups a tendency for a better five-year EFS in the more intensive treatment arms (SR: 0.77 (VAIA) vs. 0.61 (VACA) ($p = 0.32$); HR: 0.61 (EVAIA) vs. 0.50 (VAIA) ($p = 0.3577$)). Further analyses showed a significant better outcome for pts with small ET (0.69 (<100 ml) vs. 0.61 (100–200 ml) vs. 0.51 (>200 ml), $p = 0.0017$) and for pts with good histological response to chemotherapy (0.72 (<10% viable tumour cells) vs. 0.48 (>10% viable tumour cells), $p = 0.0001$). Multivariate Cox regression identified the histological response to chemotherapy as most important risk factor, compared to age, TV, and primary tumour site (risk ratio: 2.39 vs. 1.49 vs. 1.43 vs. 1.62). A further prognostic factor is the modality of local therapy. There is a significant advantage for surgical procedures with or without radiotherapy (five-year EFS: 0.65 (surgery +/- radiotherapy) vs. 0.47 (radiotherapy), $p = 0.0001$), with a higher incidence of local (9% vs. 3%) and combined relapses (10% vs. 3%) in the group of irradiated ET pts. According to toxicity, WHO grade 4 neutropenia was highest in pts randomised for EVAIA, but this was not associated with a higher incidence of severe infections or secondary malignancies.

In conclusion the results regarding randomised treatment strategies at present show no statistical significance. Longer follow-up is needed for definitive results. The preference for surgical procedures has improved the safety of local control.

*Supported by Deutsche Krebshilfe, EU-BIOMED, and Sabrina Forschungsfond.*

**Ewing's sarcoma: the Italian experience and strategy**

PIERO PICCI, GAETANO BACCI, STEFANO FERRARI & MARIO MERCURI
*(For the Italian Sarcoma Group)*

From 1972 to 1997, 494 patients with non-metastatic Ewing's family tumors, comprising Ewing's sarcoma, Askin tumor and peripheral neuro-ectodermal tumor (PNET), were treated according to 5 protocols. The strategies of chemotherapy treatment changed over the years: from 1972 to 1988, a 3-drug regimen with vincristine (V), doxorubicin (A) and cyclophosphamide (C) gave significantly worse oncologic results (10-yr DFS 28%) than those obtained with a 4-drug regimen with V-A-C and actinomycin D (Act) used either as adjuvant (10-yr DFS 52%) or as neo-adjuvant chemotherapy (10-yr DFS 46%). From 1988 ifosfamide (I) and etoposide (E) were added to the V-A-C-Act regimen. In a study conducted between 1988 and 1991 Ifosfamide and Etoposide were used in the maintenance phase added to V-A-C-Act without improving oncologic results (5-yr DFS 54%). In the subsequent study Ifosfamide was used from the induction phase added to V-A-C-Act, and Etoposide was used in the maintenance phase. With this protocol, 3-yr DFS was 78%. Strategies for local control also changed: in the first two adjuvant protocols radiation therapy (RxT) alone was used in 67%, and 49% of patients respectively, whereas in the last neoadjuvant protocol 72% of patients underwent surgical treatment. From 1993, a separate protocol for high-risk tumors (pelvis, axial skeleton and all sites metastatic) was activated.

A retrospective analysis on all patients, showed the importance of surgical treatment in local control and the strong prognostic significance of tumor necrosis induced by primary chemotherapy in surgically treated patients. In the first protocols the axial location of the tumor was an adverse prognostic factor, but in the last protocol axial and extremity tumors did not show a significantly different oncologic outcome.

These data, and, particularly, the grade of chemotherapy induced necrosis, were used to define the different risk groups and the ongoing protocols.

**Abstracts—Posters**

**Long-term outcome of patients with malignant fibrous histiocytoma**

D. ZURAKOWSKI, M. PEIPER, C. ZORNIG, W.T. KNOEFEL, & J.R. IZBICKI
*(Department of Surgery, University Hospital, Hamburg, Germany and Harvard Medical School, Boston, Massachusetts)*

*Purpose:* To determine outcome of patients with malignant fibrous histiocytoma (MFH) of the extremities and trunk.

*Patients:* From a consecutive series of 417 patients with soft-tissue sarcoma operated on 1988–98, 86 patients had MFH (45 males, 41 females). Mean ± standard deviation age was 56 ± 16 years. Mean follow-up was 5.2 ± 3.4 years for all patients; 7.5 ± 2.6 years for the survivors.

*Methods:* Retrospective cohort study. Survival, freedom from LR and metastases were determined by Kaplan–Meier method with 95% confidence intervals (CI). Cox model and hazard ratio (HR) was used to determine risk factors. Covariates included age, gender, location, tumor grade, size, depth, resection quality, metastases or lymph nodes at presentation.

*Results:* Forty-one patients died (36 by tumor), 36 had LR, 30 developed metastases. Kaplan–Meier disease-free survival (95% CI) was 90% (85–95) at 1 year, 65% (55–75) at 5 years, 52% (40–64) at 10 years. Multivariate risk factors were tumor size (HR = 4.7), depth (HR = 3.2), and lymph nodes (HR = 5.8) (all $p<0.01$). Freedom from LR was 75% (68–83) at 1 year, 60% (50–70) at 5 years. Resection quality was the only multivariate risk factor of LR (HR = 6.8, $p<0.001$). Freedom from DM was 82% (75–90) at 1 year, 63% (53–73) at 5 years and 10 years. The only risk factor of DM was tumor depth; patients with subfascial tumors fared worse (HR = 2.2, p = 0.007).

*Discussion:* MFH patients with deep or large tumors have worse outcome, particularly given the development of DM during follow-up. These patients may benefit most from adjuvant radiation.

**Perineal soft tissue sarcoma: a retrospective analysis of 22 patients.**

F. DURAME*‡, A. CALVACANTI*‡, A. LE CESNE*, C. LEPECHOUX*, P. TERRIER*, D. VANEL* & S. BONVALOT*‡

*(Department of Surgical Oncology‡, Sarcoma Unit* Institut Gustave-Roussy Rue Camille Desmoulins. Villejuif France)*

*Objective:* to clarify particularities of perineal soft tissue sarcoma.

*Method:* retrospective analysis of 22 patients with a median age of 34 years. Stromal tumours, paediatric sarcoma and pelvic tumours with perineal extent were excluded.

*Results:* Time to diagnosis was 6 months after non-specific signs. Ischio-rectal topography was the most frequent localisation (27%). 68% were high-grade sarcoma. Rhabdomyosarcoma (36%) and leiomyosarcoma (22%) were the main histological subtypes. Lymph-nods were involved in 27% cases. 23% of patients had synchronous metastasis. 86% of patients had inadequate first surgery. Surgical re-excision allowed an R0 resection in 90% patients (tumours completely surrounded by uninvolved tissue). Local control is influenced by quality of excision (R0 versus R1 R2). The median overall survival (25 months) is influenced by initial stage, grade and depth of the tumour.

*Conclusion:* Primary perineal sarcoma is a rare aggressive entity with dismal prognosis. An initial biopsy is necessary to avoid inadequate surgery. Re-excision allows an R0 resection in 90% cases but is associated with more sequels than performed initially. Impact of induction chemotherapy must be evaluated.

**Provincial variations in soft tissue sarcoma (STS) treatment and outcomes**

C. DE GARA

*(Cross Cancer Institute, Edmonton, Canada)*

*Purpose:* This project examines intra provincial variations in Soft Tissue Sarcoma (STS) Treatment and Outcomes. Level I evidence favours limb sparing surgery plus postoperative radiotherapy and single agent adjuvant chemotherapy. Institution specific level V evidence tend to advocate numerous alternatives

*Patients/Subjects:* All STS cases diagnosed in Alberta between 1990 and 1998 were identified from the Alberta Cancer Registry, a population based cancer registry. Population-based data may be less prone to selection, referral and treatment biases inherent in single center series. Healthcare in Alberta is universal, government funded and single tiered. Alberta has 2 regional cancer centres (north and south). Although the complex multi-disciplinary nature of STS ensures all cases are managed at regional cancer centres, treatment and outcomes variations may exist. Variations in patient demographics, tumour characteristics, treatment and survival were examined.

*Results:* There were 327 STS diagnosed, 61% were > 50 years, with MFH (29%), leiomyosarcoma (14%) and myxoid liposarcoma (8%) being the commonest morphologies and lower limb (43%), upper limb (15%) and pelvis (14%) being the commonest sites.

*Discussion:* Potentially important STS treatment differences were observed across Alberta. These may translate into short term but not long-term survival differences. Population based research may have a significant role in STS management.

**Deep soft tissue sarcomas of the extremities. Management analysis of 120 patients**

Y. KOLLENDER, J. BICKELS, O. MERIMSKI, J. ISAKOV, G. FLUSSER, A. NIRKIN, & I. MELLER.

*(The National Unit of Orthopedic Oncology. Department of Surgery B. Sourasky Medical Center, Tel Aviv Israel)*

The national Unit of Orthopedic-Oncology, Tel-aviv Medical center, Tel-aviv, Israel Deep soft tissue sarcomas of the extremities often present as a big mass near the major neurovascular bundle. For that reason in some cases free marginal resection cannot be achieved without causing major morbidity to the patient. New neoadjuvant modalities as chemotherapy or regional therapy (I.L.P) are given to patient in attempt to achieve the goal of safe resection and avoid major disability or high rate of local recurrence.

*Materials and methods:* From 1.1992 to 12.1997, 120 patients were diagnosed with deep soft tissue sarcomas. There were 67 males and 53 females whose age ranged from 2 to 90 (median, 56 years). 96 tumours presented in the lower extremity and 24 in the upper limb. 90% of the tumours were graded as high grade histologically. 53% of the tumours were adjacent to the major neurovascular bundle. Tumours that were extremely large or close to the neurovascular bundle were treated with neoadjuvant chemotherapy or isolated limb perfusion. Wide surgical resection was attempt in all cases. Radiation therapy was added post surgery in most cases. Adjuvant chemotherapy was given according to the treatment protocols.

*Results:* 22 patients were given neoadjuvant chemotherapy and 24 patients underwent I.L.P. In 78 patients wide margins were achieved, radical margins in 11 (7 amputations) and 28 marginal margins. Adjuvant chemotherapy was given to 23 patients and radiation to 77 patients. 16 patients had local recurrence. At most recent follow up 28 patients died of systemic disease (23%). Only 3 patients (14%) from the neoadjuvant chemotherapy group died from systemic disease.

*Conclusions:* The use of neoadjuvant modalities as chemotherapy or Isolated limb perfusion, attempted wide excision, and adjuvant chemo and radiation therapy achieve low recurrence rate in deep soft tissue sarcomas of the extremities. Neoadjuvant chemotherapy may improve survival.

**A 20-year review of haemangiopericytoma in Auckland, New Zealand**

M.F. BORG & C.S. BENJAMIN

*(Royal Adelaide Hospital, Department of Radiation Oncology, North Terrace, Adelaide, South Australia)*

| | | Tumour grade | | | Treatment | | | Survival |
|---|---|---|---|---|---|---|---|---|
| Low | High | unstated | CT+RT+ | only | 3 year5 year | SxRT+SxSx | | |
| **Northern Alberta** | 27% | 41% | 32% | 4% | 29% | 46% | 68% | 63% |
| **Southern Alberta** | 24% | 17% | 59% | 37% | 12% | 30% | 75% | 62% |
| **p value** | ns | ns | sig | sig | sig | sig | sig | Ns |

*Purpose:* The records of all patients registered with a histological diagnosis of haemangiopericytoma in Auckland between 1970 and 1990 were reviewed retrospectively, with the aim of determining the natural history of the disease and the response to various treatment modalities. A total of 24 patients were identified, having a median age of 45 years.
*Patients/Subjects:* Twenty-one patients (87.5%) underwent surgery; the remaining three were deemed to be unfit for surgery. Seven patients (29%) were treated with surgery alone; nine (37.5%) received a radical course of radiotherapy and three (12.5%) received palliative radiation therapy for pain relief and/or dyspnoea. Five patients (21%) received chemotherapy during the course of their disease.
*Results:* Eight of the 24 patients (33%) were alive and disease free, 13 (54%) having died and three (13%) being lost to follow-up. Seven patients (29%) died as a result of metastatic disease. Three of the seven (43%) who were treated with surgery alone are known to be alive and disease free. The three patients who had received palliative radiotherapy, died within 2 months of completing the latter treatment. Five of the nine patients (56%) receiving a course of radical radiotherapy are alive and disease free at present. No local recurrence was noted following surgical excision and postoperative radical radiotherapy, whilst eight (67%) of those initially treated by excision alone developed recurrent disease. None of the patients treated with chemotherapy obtained significant palliation.
*Discussion:* Results suggest that adequate surgical excision followed by postoperative radiotherapy is effective in controlling haemangiopericytoma and that metastatic disease is at present invariably fatal. The role of chemotherapy needs further investigation.

**Multiple myxoid liposarcomas or metastases? P53 and LOH analysis.**

A.N. VAN GEEL, M.A. DEN BAKKER* & W.N.M. DINJENS*
*(Dept of surgical oncology and *pathology, University Hospital Rotterdam/Daniel den Hoed Cancer Center, Rotterdam, The Netherlands)*

*Purpose:* A study of multiple deposits of liposarcoma in an single patient to determine if these deposits are primary lesions or metastases.
*Patient:* A 32 year old female was seen in our clinic with a recurrent liposarcoma in the knee and deposits in the left paracolic region, right ovary, hilus of the liver, right kidney, left breast and anterior mediastinum. After doxorubicin (7 cycles) radical resection of all deposits was achieved by sternolaparotomy. Two years later a retroperitoneal recurrence was removed and currently, 4 years after initial presentation she is suffering from am retroperitoneal and mediastinal recurrence.
*Results:* DNA was extracted from formalin-fixed, paraffin embedded tumour tissue from the various recurrences and analysed for the presence of p53 mutations and loss of heterozygosity (LOH) using 20 polymorphic markers.
*Discussion:* Because soft tissue metastases from myxoid liposarcoma are highly uncommon it was thought that this could be a case with multiple primary liposarcomas. We sought to establish the relationship between the various liposarcoma deposits with molecular techniques. No p53 aberrations were found and LOH was not observed in any one of the tumour samples.
We conclude that LOH-analysis is not a suitable technique to determine whether metastases in soft tissue from a liposarcoma really are metastases and not synchronous primary liposarcomas. Secondly, the absence of LOH indicates that other, possible more specific genetic events underlie myxoid liposarcomas.

**Ossifying fibromyxoid tumour of soft tissues**

S. HOLCK[1], J.G. PEDERSEN[2], T. ACKERMANN[3] & S. DAUGAARD[4]
*([1]Department of Pathology, Hillerød Hospital, [2]Department of Orthopedic Surgery & [4]Department of Pathology, University Hospital, Cph, [3]Department of Surgery, Frederikssund Hospital, Copenhagen, Denark)*

*Purpose:* Ossifying fibromyxoid tumour (OFMT) is a rare lesion of soft tissues. Thus, in our institutions, each receiving some 30.000 histological specimens per year, we have encountered merely one such case each since its original description in 1989. We wish to present these 2 cases, which show features typical of this distinct entity, but also add unusual details.
*Patients:* #1: 55-year-old female with a tumour of the back and right axilla. The tumour measured 12 cm, had a heterogeneous cut surface regarding consistency and colour. #2: 62-year-old female with a tumour at the base of left thumb. The tumour measured 1.7 cm and had a lobular contour. The general histological pattern of both tumours featured bland, round to plump spindly cells arranged in lobules. Mitoses were few in #1; in #2 up to 4 mitoses/HPF were counted. Lesional cells were Vim+ and S-100+, #2 also desmin+. Bony spicules were seen centrally as well as peripherally in #2. Electron microscopy showed cytoplasmic projections with intermediate filaments and focal duplication of basement membranes. Fibronexus-like structures were seen in #2.
*Discussion:* The morphology of both cases was compatible with OFMT, but displayed in addition following unusual traits: #1: gigantic size, ossification confined to central portions—#2: peripheral location of the tumour, high mitotic index, presence of fibronexus-like structures. The latter is characteristic, though not specific, of myofibroblasts, and thus may provide information as to the histogenesis of OFMT. Correspondingly, a myofibroblastic derivation has previously been proposed, though most authors favour a neural origin.

**Soft tissue sarcomas of the head and neck**

C. LAJER[1], S. DAUGAARD[2], H.S. HANSEN[3], J. KIRKEGAARD[1] & M. CHRISTENSEN[1]
*([1]Dept.s of Otorhinolaryngology and [2]Pathology and [3]Oncology, Rigshospitalet (University Hospital), Copenhagen)*

*Purpose:* To report our experience with malignant and borderline soft tissue tumours (STT) of the head and neck region 1977–2000, with the additional intention of establishing a foundation for a future database of these lesions.
*Material and methods:* So far, 34 records from adult patients have been identified from the archives and pathological specimens reviewed.
*Results and discussion:* Three patients were excluded for having sarcomatoid carcinomas. Remaining were 20 men and 11 women, median age 56 years. The most common location was the nasal cavity and paranasal sinuses, followed by the maxilla and alveolar ridge. After review, which changed the diagnosis in 9 cases (26%), the most common histological diagnoses were leiomyosarcoma (6 cases), synovial sarcoma (4) and hæmangiopericytoma (4). Like others, we saw no liposarcomas, but a relatively high proportion of borderline malignancies. 28 patients underwent primary surgery with curative intent, while 3 patients had a biopsy only. 8 received post-operative irradiation and 1 patient received irradiation as sole treatment. Only 4 patients received adjuvant chemotherapy. Mean follow up time was 28 months (range 1–127 months), with 19 patients (61%) recurrence free; 8 of these had borderline malignancies (4 hæmangiopericytomas, 3 hæmangioendotheliomas, 1 aggressive fibromatosis). 9 (29%) had died from their disease—5 of them with distant metastases and only 2 with lymph node metastases.
*Conclusion:* Head and neck STT differ in their composition from those of other sites. Because of their rarity, they present a diagnostic as well as therapeutic challenge. Treatment should be centralized and clinical databases established in order to collect experience, preferably in intergroup trials.

anatomy of the neck. Therefore, other treatment methods should be considered for fibromatosis of the neck.

**The treatment of aggressive fibromatosis at a specialized centre.**

C.A. GRAFTON*, K. GODDARD*, C. BEAUCHAMP#, K. MORTON# & M. KNOWLING*
*(*Vancouver Cancer Centre, British Columbia Cancer Agency (BC-CA), and #Vancouver General Hospital, B.C., Canada)*

*Purpose:* At BCCA aggressive fibromatosis is treated using the same oncological principles as sarcomas. The following is an audit of this approach compared with patients treated elsewhere and seen later in the course of the disease. Patients over 16 years old seen at BCCA from 1980 to 1993 were reviewed.
*Method:* All new patients had clinical examination and CT or MR scans prior to biopsy. Patients with a biopsy or excision performed elsewhere were also scanned. All pathology was reviewed at BCCA. Treatment was by wide excision with radiotherapy when either surgical margins were narrow or to enable limb sparing. For patients previously resected and found to have a marginal excision, a re-excision of the surgical bed was performed and treatment considered as of BCCA.
Results: A total of 48 patients were seen and the disease specific survival is 46/48 (92%). The disease-free survival is 48% at 5 years and 90% of relapses were within 2 years. Of patients treated at BCCA 3/13 relapsed against 26/35 treated elsewhere. Of patients treated with radiotherapy 2/12 relapsed against 27/36 without radiotherapy.
*Discussion:* The indication is that a specialized centre may attain better local control and radiotherapy may have a central role. We have not assessed the toxicity and resultant function from this approach to a disease, which is rarely fatal.

**Fibromatosis of the neck: a review of 18 cases**

C.K. HUANG, S.D. NELSON & T. CALCATERRA
*(University of California Los Angeles Medical Center)*

*Purpose:* Fibromatosis is a histologically benign, clinically aggressive tumor with a tendency to invade adjacent structures and locally recur. Fibromatosis of the neck is challenging as nearby important structures increase morbidity and make surgical excision difficult. We report a series of fibromatosis of the neck and discuss the challenges in its treatment.
*Patients:* The study consisted of 18 patients with fibromatosis of the neck seen at our institution. The clinical data was collected retrospectively from patient charts and all slides were reviewed by one pathologist.
*Results:* Patient age ranged from 20–72 with a female to male ratio of 13 to 4. All patients presented with mass, about half (n = 9) had accompanying pain, and a few had decreased mobility or Horner's syndrome. All patients underwent an attempt at a wide surgical excision. Gross margins could not be obtained in 5 patients whose tumor invaded the spine or base of skull. Microscopically negative margins were obtained on only three patients. Three patients received adjuvant radiation therapy. There was an overall recurrence rate of 47% (n = 8) after an average disease free interval of 16 months. Recurrent disease was treated with re-excision, re-excision with radiation, or chemotherapy.
*Discussion:* There is no consensus on the best treatment for fibromatosis of the neck. Though the current recommendation is a wide surgical excision, it cannot be shown to eliminate disease or prevent recurrence. A radical surgery is often limited by the

**Soft tissue sarcoma of the extremities and trunk—outcome of patients treated within 6 years under the same strategy.**

M. PEIPER, C. ZORNIG*, D. ZURAKOWSKI, W.T. KNOEFEL, C. BLOECHLE, E.G. ACHILLES & J.R. IZBICKI
*(Departments of Surgery, University Hospital Hamburg and *Israelitisches Krankenhaus, Hamburg, Germany)*

*Introduction:* Most outcome studies to determine the effectiveness of soft tissue sarcoma (STS) therapy span a long period of time and involve several different therapeutic strategies.
*Methods:* From March 1988 to March 1994, 101 patients underwent surgery for primary STS of the extremities or trunk. All patients were operated on by the same surgeon. Early re-excision was made after inadequate primary resection. Stepwise logistic regression was used to identify the important predictors of death. Cox regression models and Kaplan-Meier curves were used to assess time-related survival.
*Results:* Mean age of patients was 48 (range 15–84) years, 50% were female. The STS was located at the proximal extremity in 61% of all patients; 23% of the sarcomas were at the trunk, and 16% were distributed in the distal extremities. In 77% of patients, a resection with wide margins (R0) was achieved. In cases of R1 (n = 19) and R2 (n = 4) patients either denied reoperation or a palliative resection was performed. Postoperative radiation therapy and chemotherapy were administered in 44% and 17% of all patients, resp. The overall survivorship was 83.2% (mean follow-up 36 months). The survival rate was 90% at 1 year and 84% at 3 years. Multivariate risk factors influencing survival were tumor grade ($p = .02$), positive regional lymph nodes ($p<.01$), and location ($p = .03$). Local recurrence was not associated with increased mortality ($p = .18$). Patients with positive regional lymph nodes ($p<.001$), distant metastases ($p<.001$), and G3 tumors ($p<.01$) had shorter survival times.
*Conclusions:* Improvements in survival of patients with STS can be achieved with the use of complete excision within the anatomical compartment at initial surgery. Radiation therapy shall be performed in all cases of R1/R2 resections. High-risk patients without wide resection margins and G3 tumors may benefit most from this strategy.

**Central nervous system metastasis from sarcomas: still an unresolved problem**

A. COMANDONE, A. BOGLIONE, O. DALCANTON, S. CHIADÒCUTIN, C. OLIVA & P. BERGNOLO.
*(Oncologia, Ospedale Gradenigo, Torino, Italy)*

Soft tissue and bone sarcomas rarely cause CNS metastasis (<1%). We reviewed 19 cases of brain and medullar metastasis between 1990 and 2000. There were 10 males and 9 females, median age 48 (17–66). PS 1–3 ECOG. Histological subtypes: 7 leiomyosarcomas, 3 MFH, 2 undifferentiated, 1 clear-cells, 1 pleomorfic liposarcoma, 1 schwannoma, 1 synovialsarcoma, 1 chondrosarcoma, 1 small-cell bone sarcoma, 1 extraosseous Ewing sarcoma. The two last determined medullar metastasis. Primary sites were: 7 visceral organs, 9 extremities, 2 trunk, 1 vertebral body. 15 pts had a high-grade sarcoma. 9 pts had multiple brain mets and 10 unique. All patients were heavily pre-treated: all received surgical treatment of primary tumour and 7 adjuvant chemotherapy (CT). All 19 patients were treated with CT when disease relapsed, 7 received a second-line therapy, 6 had a third-line. Median time to relapse was 30 months (11–54). 16 patients received palliative CNS radiotherapy (RT) (15 Gy). Five patients had platinum-based CT. All patients had anti-oedema therapy. Median survival from diagnosis of CNS mets was 4.1 months (2–10). No responses were recorded. CNS mets from sarcoma have a poor prognosis, with very short survival. No significant responses can result after CT or RT. Moreover CT and RT do not seem to offer better results than best supportive therapy.

**National retrospective study of radiation-induced soft tissue sarcoma in the netherlands.**

E.A. BAARTMAN*, A.N. VAN GEEL*, F. VAN CO-EVORDEN, J.W. COEBERGH & F.E. VAN LEEUWEN
*(*Dept of surgical oncology and radiotherapy, University Hospital Rotterdam/Daniel den Hoed Cancer Center and the Dutch Soft Tissue Sarcoma Group)*

*Purpose:* To evaluate the occurrence of soft tissue sarcoma (sts) after radiation therapy and to investigate treatment strategy and prognosis.
*Patients and methods:* In this Dutch multi-institutional study records of 101 (preliminary) patients were retrospectively reviewed. Patients were eligible if they had a diagnosis of STS that occurred after sufficient latency following radiation therapy for a histologically proven distinct primary tumour and originated in the vicinity of the irradiated area. The investigated parameters included age at diagnosis, type of irradiation, dose delivered, tumour histology, anatomic location and interval between these tumours, treatment of the sarcoma, histology and follow-up.
*Results:* Preliminary results showed that the median age at the time of diagnosis of the primary was 47.3 years (11–86) and the median interval between irradiation and diagnosis of the sarcoma was 11.2 years (2–38). The median dose delivered to the primary was 47.3 Gy (16–75). Seventeen patients had developed a STS on the edge of the radiation field. Nineteen patients were seen with an angiosarcoma of the breast after breast cancer. Survival after diagnosis of sarcoma was generally poor (median 29 months).
*Discussion:* The induction of soft tissue sarcoma after radiation therapy is a rare but serious complication. Nowadays follow-up is sufficient to evaluate the different aspects of postradiation STS as will be presented in this study and side studies as case control-and dose deliverance studies are in progress.

**Gastrointestinal stromal tumours**

A.M. MOGENSEN, S. HOLCK* & S. DAUGAARD
*(Dept. of Pathology, Rigshospitalet, Copenhagen & *Dept. of Pathology, Hillerød Hospital, Hillerød, Denmark)*

*Purpose:* The knowledge that gastrointestinal stromal tumors (GIST) usually express the c-kit proto-oncogene product, a transmembrane tyrosine-kinase receptor (CD117), has now established them as a distinct entity. Hence we found it of interest to review our cases.
*Methods:* Pathological review of GISTs from the period 1990–2000 with supplementary immunohistochemistry.
*Results:* 31 cases were found, of which the first was registered in 1994, the majority appearing after 1997. 15 were females, 16 males, aged 42–88 years with most patients older than 55 years. 13 cases were malignant, 5 of borderline malignancy and 13 benign. One was a GANT, one presented as liver metastasis only, and a patient with neurofibromatosis had multiple benign GISTs.14 tumors were located in the stomach, 9 in the small bowel, 1 in the rectum and 6 in the abdomen. All tumors except one expressed CD117 and 23 tumors CD34. 30 were vimentin, 5 actin, 2 desmin and 25 bcl-2 positive. HMB45, CK, S-100 and CD99 were negative, NSE often inconclusive. EM, performed in 5 cases, revealed no specialized cell type. Some 100 other mesenchymal gastrointestinal tumors were encountered, but not yet reviewed. Of these more than half were leiomyomatous, evenly distributed between benign and malignant, the remainder a smaller number of fibrous, neurogenic, histiocytic, vascular and un! classifiable benign/malignant tumors.
*Conclusion:* Our material confirms the high frequency of CD117-positivity, justifying GIST to be diagnosed as a separate entity, thereby making possible a specific modality of postoperative treatment. We intend to complete the study by collecting clinical information, hoping to learn more about this tumor's biology.

**PET and MRI co-registration in pre- and post treatment evaluation of bone and soft tissue sarcoma.**

H.W. HENDEL, A. EIGTVED, M. NOWAK, K.E. JENSEN, S. DAUGAARD, A. KRARUP–HANSEN, J.G. PEDERSEN, D. HOVGAARD, L. DANBORG & B.H. PEDERSEN
*(On behalf of the Sarcoma Group, Rigshospitalet, Copenhagen University Hospital, Denmark)*

*Purpose:* Functional characterization of sarcomas by FDG-PET can provide complementary information to MRI and CT. This may prove useful in more accurate localization and evaluation of the tumour. Due to the heterogeneity of sarcomas, PET/MRI coregistration could provide optimal dataset for guidance of biopsy-procedures. Furthermore, image fusion with PET and MRI can provide detailed anatomical localization of the functional changes in the tumour region.
*Methods:* Until now, five patients with primary sarcomas have been included. Two have been reinvestigated after chemotherapy. A regional FDG-PET scanning for the coregistration was performed with the extremity fixed in a mould, also used during MRI. A surface-based fitting algorithm was used for coregistration the PET transmission scan to the MRI data. The emission scan was subsequently projected on the MR scan. From the PET data we calculated the Standardized Uptake Value (SUV), representing the degree of metabolic activity.
*Results:* PET and MRI agreed in three sarcomas (synovial sarcoma grade II, malignant peripheral nerve sheath tumour grade III, myxofibrosarcoma grade III) regarding morphology and metabolism. Two expressed homogeneity, one heterogeneity. However, in two heterogeneous sarcomas (high grade surface osteosarcoma, high grade osteosarcoma), MRI indicated a larger degree of tissue involvement than suggested by PET, probably due to the difficulties in differentiating reactive tissue from malignant. Post-treatment imaging revealed similar changes in tumour size. PET showed decreased SUVs.
*Discussion:* PET/MRI image fusion is feasible with potential advantages compared to conventional MRI in evaluation of sarcomas.

**Core needle biopsy to avoid open biopsy for the diagnosis of soft tissue masses (STM): a retrospective study.**

I. RAY-COQUARD, H. GHESQUIÈRES, D. RANCHÈRE-VINCE, P. BIRON, M.P. SUNYACH, M. RIVOIRE, T. PHILIP, P. MÉEUS, C. SEBBAN, P. THIESSE & J.Y. BLAY.
*(Centre Léon Bérard, Hopital Edouard Herriot, Lyon, France)*

Open biopsy is considered as standard practice for the diagnosis of soft-tissue sarcoma (STS). Core needle biopsy (CNB) is an alternative, with minimal morbidity and cost, but with a possible risk of diagnostic inaccuracy. We conducted a retrospective study of the accuracy of CNB for the diagnosis of STM.

*Method:* All adult patients in whom a CNB was performed for the diagnosis of STM between 1994 and 2000 were selected. Sensibility (Se), specificity (Sp), positive (PPV) and negative (NPV) predictive value were determined for the diagnosis of malignancy (yes or no), connective tumor (i.e. benign or malignant), sarcoma (STS), and lymphoma (NHL) by comparing CNB to the gold standard histological, molecular or clinical diagnostic tests. Total concordance was admitted when the CNB and gold standard yielded similar diagnosis including subtype (e.g. liposarcoma), a partial concordance was similar but without diagnosis of subtype (e.g. STS). All other situations were considered as discordant.
*Results:* Among 110 CNB, 7 yielded insufficient tumor material for diagnosis and 103 patients were analyzed. The final diagnosis was benign tumor (19%), STS (59%), NHL (7%), aggressive fibromatosis (5%), and carcinoma (6%). Hematoma was the only reported side-effect of CNB (6%); Median size of biopsy specimens was 19 mm (range 1–60). For all tumors, total concordance was observed in 88% (91/103), partial concordance in 7% (8/103) and discordance in 5% (5/103). Sp and PPV of CNB were 100% for the diagnosis of malignancy, STS, NHL, and aggressive fibromatosis. Se was 97% for the diagnosis of malignancy, 97% for the diagnosis of connective tumor, 92% for the diagnosis of sarcoma (100% for the diagnosis of high grade STS, 70% for the diagnosis of low grade STS), 100% for the diagnosis of NHL. NPV was 91% for the diagnosis of malignancy, 89% for the diagnosis of connective tumor, 88% for the diagnosis of STS (100% for high grade STS and 90% for low grade STS), and 100% for the diagnosis of NHL.
*Conclusion:* CNB enables to avoid open biopsy when a diagnosis of high grade STS, lymphoma or aggressive fibromatosis is obtained. CNB is less accurate in benign connective tumors or low-grade STS.

**The effect of changing diagnostical practices on the pathological subclassification of soft tissue sarcomas**

S. DAUGAARD
*(Dept. of Pathology, Rigshospitalet, Copenhagen, Denmark)*

*Purpose:* To evaluate the effect of pathological review on the diagnostic subclassification of soft tissue sarcomas (STS).
*Material and methods:* Review of available archival pathological material from patients with STS of the extremities, diagnosed in the years 1972–1995. In selected cases, immunohistochemistry was performed.
*Results:* Overall, the review has so far led to a change in diagnosis in 131 out of 273 cases (48%). Previously, the most common subgroups were malignant fibrous histiocytoma (MFH): 27%, liposarcoma: 21%, fibrosarcoma: 11%, leiomyosarcoma: 11%, and synovial sarcoma: 8%. Most were distributed fairly evenly over time, but 65% of the MFHs were diagnosed in the 3rd quarter of the period (1985–90). 84% of the fibrosarcomas and 73% of the malignant fibrous histiocytomas were reclassified, compared with only 19% of the synovial sarcomas, 18% of the liposarcomas, and 13% of the leiomyosarcomas. After review, the largest subgroup became the liposarcomas (22%), leiomyosarcomas (19%), synovial sarcomas (14%), MFHs incl. myxofibrosarcomas (10%), with 11% unclassified (NOS). Five benign lesions were identified: two cases of nodular fasciitis, two lipomas (with degenerative changes), and one aneurysmal fibrous histiocytoma. Among the fibrosarcomas were five fibromatoses (historically! considered 'fibrosarcomas grade_'). New entities were: two cases of sclerosing epithelioid fibrosarcoma and one low-grade fibromyxoid sarcoma.
*Discussion:* When using historical material, e.g. for the purpose of evaluating possible prognostic factors or new molecular biological diagnostic markers, pathological review following current diagnostical criteria should be mandatory in order to avoid statistical contamination of the study population.

**Pulmonary metastases from soft tissue sarcoma: an analysis of prognostic factors with long-term follow-up**

T.J. HIEKEN, C. SCHAEFFER & T.K. DAS GUPTA.
*(Department of Surgical Oncology, University of Illinois at Chicago, Chicago, IL, 60612, USA)*

Soft tissue sarcomas frequently metastasise to the lung, which may be the only site of metastatic disease. A proportion of affected patients may experience long-term survival. We studied 119 soft tissue sarcoma (STS) patients with lung metastases (LM) in an effort to identify factors associated with a favourable outcome. 54 female and 65 male patients, ranging in age from 18 to 83 years, developed LM at a mean of 32 months (median 11 months) from the time of initial diagnosis. Mean follow-up was 62 months for surviving patients. Overall, 3-year survival was 18% and 23 of 119 patients survived $\leq$36 months after developing LM. From numerous demographic and tumour features examined, only the development of LM > 1 year after initial diagnosis ($p = 0.002$), unilateral versus bilateral LM ($p = 0.002$), resection versus no surgical treatment ($p<0.0001$) and administration of chemotherapy ($p = 0.03$) were significant variables. The latter three parameters retained significance in multivariate analysis. For the subset of 48 patients who underwent metastectomy with curative intent, 19 survived $\geq$36 months (actuarial 3-year survival 40%). For this subgroup of patients, we identified the following markers of poor prognosis: interval to LM $\leq$1 1 year, incomplete resection and mutant p53 expression, assessed by quantitative ELISA assay, by the metastatic tumour, suggesting that such tumours are biologically aggressive. These data confirm that long-term survival may be achieved for a subset of patients with pulmonary STS metastases, predominantly those with mutant p53-negative unilateral metastases who undergo complete surgical metastasectomy.

**Pulmonary metastases in soft tissue sarcoma**

M. PEIPER, W.T. KNOEFEL, C. BLOECHLE, A. HEINECKE, E.G. ACHILLES & J.R IZBICKI
*(Department of Surgery, University Hospital Hamburg, Germany)*

*Purpose:* The lungs compromise a predilection site for pulmonary soft tissue (STS) metastases.
*Methods:* A retrospective analysis of all patients operated on pulmonary metastases of STS between 1988 and 1999 was performed. Patients and tumor characteristics as well as surgical and pathological results were evaluated. RESULTS: 52 Patients (31 female, 26 male) with a median age of 44 (18–75) years were operated. Primary tumors were leiomyosarcomas (n = 13, 23%), MFH (n = 10, 19%), malignant peripheral nerve sheath tumour (n = 6, 13%) and 23 tumors of 11 other entities. 42% of patients were already treated with primary tumors in our institution. Primary tumors were located subcutaneously in 12 patients, subfascially in 25 patients and in 15 patients in parenchymatours organs. 50% of primary tumors were poorly differentiated, while 30% were moderate and 20% were well differentiated. 29% of primary tumors were resected achieving wide margins (R0), 81% R1. In 12.5% of patients distant metastases were present at initial diagnosis. In 40 patients, chemotherapy were administered (not in randomized trials), 26 of these after diagnosis of pulmonary metastases. In 28 patients local recurrence occurred, in 25 of these before or simultanously with pulmonary metastases. Patients were operated using thoracotomy or sternotomy, no patient was operated using thoracoscopy. Up to 4 operations and up to 38 pulmonary tumors were resected. At the end of follow-up, 14 patients are alive, while 38 died due to tumor disease. Mean survival time was 36.5 months and 19.5 months after metastasectomy. No significant statistical parameters were found associated with reduced survival.
*Conclusion:* Though most patients will eventually die due to tumor disease, some curations are noted and overall survival is better than in most other malignant diseases. Even recurrent metastasectomy may be indicated in selected patients.

**Tikhoff—linberg operation and major resections of the shoulder girdle in sarcoma patients**

M. PILER & J. NOVAK
*(Department of Surgical Oncology, Institute of Oncology Ljubljana, 1000 Ljubljana, Slovenia)*

*Background and objectives:* This study was undertaken to verify the clinical results of limb-sparing Tikhoff-Linberg procedure and major resections in patients with malignant bone and soft-tissue sarcomas of the shoulder girdle treated at our Institute.
*Patients and methods:* From 1980 to 1999, 26 patients with malignant bone and soft tissue sarcomas of the shoulder girdle were treated with a limb-sparing surgical procedure at the Department of Surgical Oncology of the Institute of Oncology in Ljubljana. Eighteen patients underwent Tikhoff-Linberg procedure, and 8 had major resection of the bone and soft tissue. Of 26 patients 14 were females and 12 males with the age range 14 to 71 years (median 40 years). Sixteen patients had bone and 10 soft-tissue sarcomas. Five patients had chemotherapy, 2 postoperative irradiation and 3 patients chemotherapy and irradiation. The follow-up period in limb-sparing group ranged from 4 months to 19 years 9 months (median 6 years 4 months).
*Results:* Fourteen patients (56%) are currently alive and desease-free, with the follow-up from 1 year 3 months to 19 years (median 7 years 6 months); 2 died of other deseases, 9 died of sarcoma (4 of metastases and 5 of metastases and simultaneous local recurrence) at a mean (SD) interval of 15 months after surgery (range, 6–25 months). One patient was lost from follow-up 6 months after surgery; he was not resident of Slovenia. Of 25 patients, 2 developed local recurrence without metastasis 6 months and 4 years 3 months after surgery (median 2 years 4 months). The bone was reconstructed in 6 patients (prosthesis in 3, vascularised fibular graft in 2, and intraoperative extracorporeal autogenous sterilised bone graft in one). In 14 alive patients, hand-elbow function is excellent in 7, good in 6, and fair in 1 patient.
*Conclusion:* Classical or modified Tikhoff-Linberg operation is a suitable limb sparing procedure for tumors of the shoulder girdle. A good hand-elbow function can be preserved with local recurrences occuring in 8 percent in this as well as in majority of reported series.

**Motor unit transplantation or transposition after compartmental excision of soft tissue sarcomas**

R. CAPANNA, G. BELTRAMI, P. CALDORA, D.A. CAMPANACCI, R. ANGELONI*, M. INNOCENTI*, G. LAURI* & M. CERUSO*
*(Department of Orthopedic Oncology—*Center of Reconstructive Microsurgery, C.T.O., Florenze, Italy)*

*Purpose:* In order to obtain wide surgical margins in soft tissue sarcomas of the limbs, the sacrifice of the entire muscle compartment can be requested, with consequent function loss. After such a case, a functional reconstruction can be done by a reinnervated free muscle transplantation (motor unit transplantation) or by a pedicled muscle transposition (motor unit transposition).
*Subjects:* From 1992 to 1999, 11 patients affected by soft tissue sarcomas (8 high grade and 3 low grade), after compartment muscle excision underwent reconstruction with motor unit transplantation or transposition. All patients but 1 had been previously treated by inadequate excision. In 6 cases the motor unit consisted of a reinnervated free muscle (4 latissimus dorsi pro quadriceps, 1 latissimus d. pro extensor compartment of the leg and gracilis pro extensor compartment of the forearm). The remaining 5 cases were pedicled muscle transpositions (latissimus d. pro deltoid).
*Results:* At a mean follow-up of 47 months (min 6; max 72), 8 patients showed a satisfactory functional results (MSTS); 1 had major complication and failed postoperatively (vascular failure of the free flap); 2 were rated as unsatisfactory (after postoperative radiation therapy).
*Discussion:* Despite the difficult surgical technique, after compartment muscle defect, motor unit transplantation or transposition represents the only functional reconstruction. In case of necessity of radiation therapy, preoperative radiation therapy should be preferred for not impairing the flap's viability.

**Major limb amputation in the treatment of extended sarcomas of the extremities**

T. STREICHERT, K.A. GAWAD, M. PEIPER, C. ZORNIG & J.R. IZBICKI,
*(Departments of Surgery, University Hospital Hamburg)*

*Objective:* Curative resection of extended sarcomas of the proximal extremities can often only be achieved by major amputations due to infiltration of vital structures.
Material and Methods: 15 patients (10m:5f) with a mean age of 55 (25–81) years were treated by either exarticulation of the shoulder (n = 3) or hip (n = 5), hemipelvectomy (n = 2) or interscapulothoracic amputation (n = 5). 2 patients had a primary manifestation of their tumour, all others had recurrences. The primary manifestation was 25 (0–212) months before and the patients had 3 (0–5) prior operations for the same disease. Curative resection (R0) was achieved in all cases. The majority of the tumours were poorly differentiated (GII: n = 6, GIII: n = 9). The hospital mortality was 6%, no major complications occurred.
*Results:* After a median follow-up (after the amputation) of 30 (2–108) months 53% of the patients were still alive. 3 Patients (20%) had died tumour related, 3 due to unrelated diseases and one of unknown cause. The median survival time was 53 (2–321) months after primary manifestation of the disease and 21 (2–108) months after amputation. One patient developed pulmonary metastases 5 months after hemipelvectomy, one patient, now disease free, developed local recurrence after exarticulation of the shoulder and received R0 resection of the shoulder girdle stump. All other patients were also free of recurrence or metastases.
*Conclusion:* Major amputation can be successful even in extended, recurrent and poorly differentiated sarcomas.

**Sciatic nerve resection: is that truly an indication for amputation?**

Y. KOLLENDER, J. BICKELS & I. MELLER
*(The National Unit of Orthopedic Oncology, Tel-Aviv Sourasky Medical Center, Sackler Faculty of Medicine, Tel-Aviv University, Tel-Aviv, Israel)*

*Background:* En bloc resection of the sciatic nerve with a malignant tumour of the pelvis or thigh was long considered an indication for an amputation because of the anticipated poor function. The authors describe the functional outcome of a group of patients who underwent a limb-sparing surgery in spite of the need to sacrifice the sciatic nerve.
*Materials and Methods:* Between 1991 and 1999, the authors treated 12 patients who underwent limb-sparing resections of a malignant tumour of the thigh, buttock, or pelvis, all of which necessitated en bloc resection of a segment of the sciatic nerve with the tumour mass. There were eight females and four males, ranging in age from 2 to 73 (mean, 54 years).
*Diagnoses:* soft-tissue sarcomas—8, metastatic bone disease—3, primary bone sarcomas—1.
*Anatomic locations:* thigh—7, pelvis—4, buttock—1. Follow-up ranged from 10 to 102 months (mean, 30 months).
*Results:* At the most recent follow-up evaluation, eleven patients were ambulatory and only one patient was wheel chair bound. Of the ambulatory patients, only five patients required a walking aid (crutches or a cane); a long-leg brace was not required by any of these patients. Although all patients had an anesthetic ipsilateral foot, none had a pressure sore or required a secondary amputation. All ambulatory patients were satisfied with the functional outcome.
*Conclusion:* Provided the femoral nerve is intact, the mere necessity to resect the sciatic nerve with a given tumor of the pelvis or thigh is not an indication for an amputation.

**Hyperthermic isolated limb perfusion with TNF-a and melphalan in advanced soft tissue sarcomas: histolopathological considerations**

Y. KOLLENDER, J. ISSAKOV, M. GUTMAN. LEV-CHELOUCHE, S. ABU-ABID, K. MERIMSKY., J. BICKELS, A. NIRKIN, G. FLUSSER, N. MARUANI, B. LIFS-CHITZ-MERCER., M. INBAR., J.M. KLAUSNER. & I. MELLER
*(The National Unit of Orthopedic Oncology. Department of Surgery B. Sourasky Medical Center, Tel Aviv Israel)*

*Background, Materials and Methods:* The specimens of 27 high grade extensive soft tissue sarcomas (STS) and 3 desmoid tumours of the extremities, after local treatment with hyperthermic isolated limb perfusion (HILP) using TNF-a and Melphalan, were evaluated for the type and extent of tumor necrosis and oilier histological local tissue changes. Limb preservation was the objective in this selected group of advanced STS's, candidates for amputation or mutilating surgery otherwise. The tumoural masses were obtained 6–8 weeks after HILP, during the definitive surgical resection of the residual tumor according to protocol.
*Results:* Typical histological changes were: cystic hemorrhagic necrosis in the center of the remaining tumor with pericystic extensive fibrosis. In 8 cases more than 90"/o necrosis was achieved. In 14 cases the percent of necrosis was between 60% and 90% (including 4 cases of 80–90"/o). In 8 cases less than 60% necrosis was obtained. No correlation was found between these histological responses and: the anatomical location of the tumor, whether the tumor was primary or recurrent, the type of previous treatment (systemic chemotherapy, radiotherapy) and it's size. Some correlation was found with: the histological type of tumor and with proximal or distal location in the limb.
*Conclusions:* This is the First serial histological description of the effect of high Dose TNF-a and Melphalan administered via HILP on the tumoural masses of limb STS. The small number of specimens and especially the variability of tumours precludes definitive conclusions from the observed correlations. Larger numbers and more homogeneity of the histologic types is needed in future series.

**Beromun® (TNF-A) and melphalan for limb salvage in advanced limb neoplasms: A new standard of care for irresectable soft tissue sarcomas**

Y. KOLLENDER*, M. GUTMAN**; D. LEV-CHELOUCHE**, J.M. KLAUSNER**; J. ISAKOV*, O. MERIMSKY*; G. FLUSSER*, N. MAROUANI*; J. BICKELS* & I. MELLER*
*(*The National Unit of Orthopedic Oncology. **Department of Surgery B. Sourasky Medical Center, Tel Aviv Israel)*

*Background:* Beromun(B) (-rTNFa) is a highly potential antineoplastic agent. However, since its systemic administration in humans resulted in a life-threatening septic shock-like syndrome, its use was abandoned
These systemic side effects were eliminated when TNF was administered via isolated limb perfusion (ILP). This method is now being used in order to prevent amputation ormutilating surgery in patients suffering from irresectable limb soft tissue sarcomas.
*Methods:* During a 7-year period, 70 pts with high grade STS underwent 81 ILPs with high dose Berornun® (3–4mg) and Melphalan (1–1.5mg/kg). There were 37 males and 33 females. The mean age was 56 years (range 14–80 years).
. 31 pts presented with recurrent and 40 with very extensive primary tumors. The tumors were located in the upper extremity in 13 pts and in the lower extremity in 57 pts. All pts were candidates for either amputation or extensive mutilating surgery. ILP was performed via the corresponding vessels proximal to the tumor. Resection of the residual tumor or tumor bed or limb was performed 6–8 weeks after ILP, and all reported responses were pathologically confirmed.
*Results:* Marked tumor softening occurred within 48 hours, and in tumors protruding through the skin, hemorrhagic necrosis was evident within 24 hours. The overall response rate was 80%. 21 pts (30%) had a complete response and 40 (57%) had a PR. in 3 pts (11%), only minimal regression was observed (stabilization of disease). Operative mortality was 3% (2 pts). Limb sparing was achieved in 82% (62/70 pts). Amputation was performed in 8 pts. Within a follow-up period of 2–76 months )median 24 m), 30 pts (43%) are dead, 31 (44%) are alive with no evidence of disease (median 49 m), and 9 (13%) are alive with disease. Local recurrence occurred in 16/60 evaluable pts (26%). Limb salvage and survival rates were not significantly different for gender, age, primary vs recurrent tumor, or tumor location.
Mortality was significantly higher for multifocal disease (73% vs 29%)(p<0.05). Limb salvage rates were also not different for the various histological subtypes. There was trend towards higher response rates in synovial and clear cell sarcomas.
*Conclusions:* The combination of Berornun® and Melphalan given via ILP appears to be effective in pts with advanced STS confined to the limb, achieving a high response rate and limb preservation.

**Primary radicalization plus radiotherapy after inadequate surgery in soft tissue sarcomas**

G. BELTRAMI, R. CAPANNA, P. CALDORA, D.A. CAMPANACCI, A. FRANCHI* & M. PERTICI**
*(Department of Orthopedic Oncology, CTO, Florenze; *Department of Pathology, Florenze **Department of Radiotherapy, Florenze)*

*Purpose:* After inadequate margins in Soft Tissue Sarcoma (STS), there is no general agreement about further treatment (surgery and or radiotherapy). This is particularly evident with no evidence of clinical or radiological disease. We report our experience, comparing two groups of patients: one, primarily observed in our Center (group A), and one inadequately operated elsewhere (group B). Both groups underwent surgery, and, when feasible, brachytherapy and external radiation therapy.
*Subjects:* From 1987 to 1999, 241 patients affected by STS of limbs have been treated in our Center: 68 where first observations (group A), 75 where primary radicalizations (group B). Ninety-eight have been excluded since affected by local recurrence (operated elsewhere) or amputated. High grade STS were represented in 68% of group A and 80% of group B while a location distal to knee or elbow was in 35% of group A and 63% of group B.

The average time of radicalization after the previous surgery was three months and viable tumoural cells were found in 45% of specimens of group B.

Free or rotational flaps, for covering the loss of substance, were performed in 20% of group A and in 44% of group B.

Brachytherapy was applied in 63% of group A and in 57% of group B, while conventional radiation therapy was performed in 79% of group A and 67% of group B.

*Results:* At an average F.U. of 54 months (5–150), 76% of patients were continuous disease free in both groups. Local recurrence occurred in 3% of group A and in 5% of group B. In group B, as far as the local recurrence rate is concerned, 6% appeared in positive specimens and 5% in negative specimens. Functional results (MSTS) were rated as satisfactory in 80% of group A and 88% of group B.

*Discussion:* After inadequate surgery of STS, surgical reprises associated to adjuvant radiation therapy showed to be efficacy in local control, even in distal location, although with high percentage of free flaps, not impairing the functional results.

**Is adjuvant radiation (RT) indicated after optimal resection for soft tissue sarcoma (STS) of the extremities?**

A. LE CESNE, K. KHANFIR, P. TERRIER, C. ALZIEU, BONVALOT, D. VANEL, T. TURSZ & C. LE PECHOUX
*(Institut Gustave Roussy, Villejuif, France)*

The impact of adjuvant RT on both local control and distant relapse is not clearly established after wide excision of extremity STS.

*Purpose:* We performed a retrospective analysis on behavior of patients (pts) who underwent a large resection (first or second resection) in our institution and received or not adjuvant RT: All histological specimens were carefully analyzed and only pts with free tumoral margins (ftm) were retained for analysis. Histopathological classification was as following: minimal R0 (mR0) resection (ftm<10mm) and optimal R0 (oR0) resection (ftm ≥10 mm).

*Patients:* from 1975 to 1996, 133 pts with a median age of 44 yrs were operated at IGR. The median tumor size was 6cm. Ninety-three pts (70%) primary resected in other centers, were reoperated and residual tumor cells (RTC) were found in 54% of pts. Sixty-nine pts (17 oR0 and 52 mR0) received adjuvant RT and 64 pts did not (54 oR0 and 10 mR0). Characteristics of pts were similar in both groups.

*Results:* Median follow-up time was 1o yrs (3 or 25). Thirty-three pts had a local relapse: 11 in the RT group and 22 pts in the control group (p 0.01). Grade and ftm are correlated to OS and adjuvant RT to RFS. A positive impact of RT on local control was only seen in pts with a mR0 resection (p = 0.005) and in pts with RTC after reexcision (p = 0.001). RT has no influence on 5 and 10 yr-OS.

*Conclusion:* optimal resection seems to be the best predictive parameter for a favorable outcome in term of local control in localized STS. Adjuvant RT is indicated in mR0 resections and in case of RTC after definitive surgery, but its role after oR0 resection has to be validated by a prospective randomized trial.

**Local control after external beam radiation therapy (EBRT) and intraoperative brachytherapy (BT) in soft tissue sarcomas (STS).**

G. SCARZELLO, *C.R. ROSSI, R. MAZZAROTTO, A. RIGON, M.S. BUZZACCARINI, *M. FOLETTO, *P. PILATI, **A. DAL PALÙ, **F. MICHIELAN & G. SOTTI.
*(Dept. of Radiation therapy, *Dept of Surgical and Oncologic Sciences, **Dept of Anestesiology Azienda Ospedale Università, Padova, Italy)*

Since 1988 we have been collecting data regarding pts. with histologically proven diagnosis of STS, treated with conservative surgery and EBRT plus intraoperative BT, in order to assess local control and late effects. In our files were registered 64 pts. with primary localization as follows: 54 extremities, 3 head-neck, 3 trunck-abdomen, 4 genito-urinary system. All pts. underwent BT with intraoperative implant up to a total dose of 15–25 Gy. 7 of them previously received 40–50 Gy of preoperative EBRT, 57 received EBRT roughly a week after BT. 4 had hyperthermic antiblastic intraartherial perfusion before operation. 5 paediatric pts. were treated only with BT. No one had considerable radiotherapy related acute toxicity. At a median follow-up of 4 years, we registered a local relapse free survival of 95.3%. No severe late effects are observed at this dose of BT. BT allows to give RT avoiding side effects related to external beams, moreover it reduces the time for the local treatment, anticipating chemotherapy if required.

**Neoadjuvant concurrent chemoradiotherapy and extremity preserving surgery in the soft tissue sarcomas of the extremities**

Y. ANACAK[1], Z. ÖZSARAN[1], D. SABAH[2], T. AKALN[3], A. MEMI[4], A. HAYDAROLU[1], G. YÜCETÜRK[2], R. ARKUN[4] & G. KANDILO_LU[3]
*(Ege University Medical School [1]Radiation Oncology, [2]Orthopedics and traumatology, [3]Pathology, [4]Radiodiagnostics–Izmir–Turkey)*

*Purpose:* This phase-II study was designed to assess the role of neoadjuvant chemoradiotherapy and extremity preserving surgery on the local control and survival of the patients having extremity soft tissue sarcomas. The histological response of the tumors to the neoadjuvant therapy was also assessed.

*Patients:* Between 1994 September—2000 May 68 cases were included to the study having the median age 44 (17–77). M/F ratio was 1.7. The diagnosis was done with tru-cut biopsy; the most common subtype was liposarcoma.

*Methods:* All cases were treated with 50.4 Gy (2Gy/day, 5 fractions/week) and 3 cycles of concurrent chemotherapy (mitomycin 8mg/m2, doxorubicin 40mg/m2, cisplatinum 60 mg/m2). One month after the end of neoadjuvant therapy wide excision was performed in 52 cases and amputation in 2 cases. 14 patients were not operated due to refusal of operation or metastatic outcome. 2–3 cycles of additional chemotherapy was given in 11 cases.

*Results:* After a median follow-up of 27 months (3–74), local recurrence was developed in 4, lymph node metastasis in one and distant metastasis in 21. Local recurrence free survival was 88.8% and distant metastasis free survival was 49.1% at 3 years. 17 cases were lost due to tumor progression; cause specific survival was 51.9% at 3 years.

*Discussion:* Concurrent neoadjuvant chemoradiotherapy in soft tissue sarcomas is feasible. Further randomized studies are needed to clarify the role of neoadjuvant radiochemotherapy on the local control and survival of soft tissue sarcomas.

**Re-excision of retroperitoneal sarcoma after inadequate initial surgery**

A. CALVACANTI*‡, A. LE CESNE*, C. LEPECHOUX*, P. TERRIER*, D. VANEL* & S. BONVALOT*‡
*(Department of Surgical Oncology‡, Sarcoma Unit* Institut Gustave-Roussy Rue Camille Desmoulins. Villejuif France)*

Re excision of limb's sarcoma after inadequate surgery finds 45 to 50% residual tumour cells.

*The objective* of this study is to determine the prevalence of residual tumour after marginal excision of retroperitoneal sarcoma (RPS). Patients with residual mass on imaging or described by the medical report were excluded.

Among the 94 retroperitoneal sarcoma operated at Institut Gustave-Roussy between November 1997 and November 2000, ten underwent systematic re-excision. Initial surgery was in these cases marginal excision of the RPS with 4 effractions of the tumour. Median age of the patients was 41 years. FNLCC grade were grade 1 n = 4, grade 2 n = 3, grade 3 n = 3. 'En bloc' resection of the initial surgical area was performed with 5 colectomy, 6 muscle's resections, 1 posterior pelvectomy, 1 omentectomy, 1 duodeno-pancreatectomy, 1 nerve's excision.

*Results:* Residual tumour was found in 7 patients (70%). Macroscopically obvious n = 3 (with 2 sarcomatosis) and microscopically n = 4. Surgical margins when residual tumour was found were R0 n = 4, R1 n = 3 (2 sarcomatosis) (UICC R classification). Postoperative treatments were: intra peritoneal chemotherapy n = 2, radiotherapy n = 3, chemotherapy n = 1.

With a median follow up of 20 months, 4 recurrences occurred; the two patients with sarcomatosis and 2 patients with initial tumour's effraction but with no residual tumour found at re-excision. One recurrence was both local and metastatic.

*Conclusions:* As limb's sarcoma, marginal excision and effraction of retroperitoneal sarcoma are prohibited. Surgery must be << en bloc >> with adjacent organs*. Marginal excision of RPS leads to high rates of residual tumours (70%) and re excision after inadequate surgery must be discussed.

*Surgical management of primary and recurrent soft tissue sarcoma of the retroperitoneum ASCO Proceedings, 1997, 1807. Bonvalot S, Dube P, Le Cesne A Terrier P, Vanel D, Genin J.

**Peritoneal hyperthermic perfusion after cytoreductive surgery in patients with retroperitoneal sarcomas and GISTS**

M. FOLETTO, C.R. ROSSI, P.L. PILATI, M. DE SIMONE, M. DERACO AND M. LISE
*(Clinica Chirurgica, University of Padua, Italy)*

*Purpose:* Retroperitoneal sarcomas and GISTS are usually a challenging therapeutical issue, with high recurrence rates. The purpose of this work was to evaluate, within a phase 1 study, the feasibility of cytoreductive surgery (CS) in combination with hyperthermic intraperitoneal intraoperative chemotherapy (HIIC) with doxorubicin and cisplatin.

*Patients and methods:* 18 out of 32 enrolled patients had recurrent or multiple retroperitoneal sarcomas/GISTS underwent HIIC with doxorubicin and cisplatin at escalating dose (5–19 mg/l and 20–43 mg/l, respectively), after CS (tumor residues < 3 mm). Loco-regional toxicity was evaluated according to modified Ozols' criteria while systemic according to WHO criteria. Follow-up controls were scheduled monthly for the first 3 months, then every three months until 18th month and then every six months.

*Results:* Post-operative complications occurred in 4 patients (2 septic shock, 1 fistula and 1 pneumothorax). Loco-regional toxicity was grade 1 in 5 patients, grade 2 in 3 and grade 4 in one that required re-operation for adhesiolysis after 20 days. Grade 1 hematological toxicity was reported in 3 patients, while persistent fever in another 3. After a median follow up of 24 months (range 12–36), 9 patients are NED (50%), 6 (33%) are AWD, 2 (11%) DOC and 1(5%) die of tumor progression. 7(39%) patients developed local recurrence and 3 of them had re-do HIIC.

*Conclusions:* HIIC in combination with CS is feasible and seems to improve local disease control in this subset of patients, with acceptable toxicity and morbidity. Large series is needed to confirm these results.

**Preoperative chemotherapy for myxoid liposarcoma**

S. KURATSU, N. ARAKI, A. MYOUI, T. UEDA, N. TAMAI, S. JOYAMA & H. YOSHIKAWA
*(Department of Orthopaedic Surgery, Kure National Hospital 3–1 Aoyamacho, Kure-city, Hiroshima 737–0023, JAPAN)*

*Purpose:* Myxoid liposarcoma(ML) has been classified as an intermediate grade tumor with a definite metastatic potential. But little is known about its sensitivity to chemotherapy. The current study reports on the clinical results and evaluates the efficacy of preoperative chemotherapy.

*Patients and Methods:* We reviewed our experience in 26 cases of ML treated at Osaka University Hospital and two affiliated hospitals from 1982 to 1997. The median follow-up was 63 months(range, 10–207).

*Results and Discussion:* There were 13 male and 13 female patients with age range from 19 to 72 years(median:46). The most common lesion was the thigh(62%). The median size of primary tumor was 10 cm in maximum dimension. Of 23 cases treated with chemotherapy, 13 cases were received doxorubicin-, cisplatin-, and ifosfamide-based chemotherapy(mean, 2 cycles) preoperatively. In 13 cases, 10 cases were evaluable for response(6 partial response, 4 no change). The surgery had intralesional or marginal excision in 1, contaminated wide (almost wide and partially marginal) excision in 5, and wide excision in 4. Among the 10 cases, 1 that underwent intralesional excision because of large tumor recurred and metastasised. At the last follow-up, 9 patients were alive with no evidence of disease, and 1 with local recurrence had died due to a lung metastasis. All of 8 cases underwent initially marginal excision only at the other hospitals recurred locally and subsequently were treated at our hospitals, whereas none had recurred and metastasised in 5 cases received preoperative chemotherapy in spite of contaminated wide excision. ML is usually intra or intermuscular, and is often large and bulky. Local recurrence is more common if inadequate(intralesional or marginal) excision has been performed. Therefore ML adjacent to bone, neurovascular structures is difficult to control locally by surgery only. We conclude that preoperative chemotherapy should be considered for patients with large, bulky myxoid liposarcoma in the neoadjuvant setting so as to minimize the morbidity of radical local therapy in responding patients.

**Orthoptic heart (ht) and heart-lung (hlt) transplantation for unresectable primary cardiac sarcomas (pcs).**

S. TALBOT[1], M.L. KEOHAN[1], L. SCHULMAN[2], N. EDWARDS[3], M. GALANTOWICZ[3], M. OZ[3], R.E. MICHLER[3], M. GINSBURG[3] & R. TAUB[3]
*(1. Divisions of Medical Oncology[1], Medicine[2], and Cardiothoracic Surgery[3], College of Physicians and Surgeons, Columbia University, New York, NY)*

*Purpose:* The prognosis for patients (pts) with primary cardiac sarcomas (PCS) is poor, especially when surgical extirpation is not feasible. The median survival (MS) is generally <10 months. Removal of all cardiopulmonary structures involved by tumour, followed by orthotopic HT or HLT has been attempted to improve long-term survival. Patients: From 1993–1999, we performed 4 heart and 4 combined heart and lung resection followed by HT or HLT in 8 pts (3 men, 5 women).

*Results:* Median age at diagnosis was 43 years (range 37–64). Of 8 pts, 6 were given doxorubicin-based chemotherapy prior to HT/HLT. Median follow up is 43 months (m) (range 5–67). Median time from diagnosis to transplantation is 7 m (range 2–25). All but 2 HT patients developed metastatic disease progression (DP). Median time to DP from diagnosis was 31 (range 28–35) and 12 m (range 10–40), and from transplantation 24 (range 20–29) and 5 m (range 0–34) for HT and HLT pts respectively. Six pts died: 3 HT and 3 HLT pts. MS from diagnosis was 53 (range 4–92+) and 39 m (range 11–57+), and from transplant were 45 (range 2–67+) and 31 m (range 5–49) for HT and HLT pts respectively. One HT pt died suddenly 3 m after transplantation without clinical evidence of disease, and 1 HLT pt died 5 m post transplant of respiratory failure after a complicated post operative course but had DP. Two pts remain alive: 1 HLT pt 47 m post POD with cerebral metastases as the only site of recurrence!, and 1 HT pt free of disease 67 m post transplant.
*Discussion:* Orthoptic HT or HLT is a technically feasible treatment for highly selected pts with localized advanced PCS. The high incidence of metastatic disease may limit the usefulness of HT or HLT.

**Use of valspodar and Doxorubicin for the treatment of P-glycoprotein positive sarcomas.**

E. CAGLIERO, N. BALDINI, S. BRETTI & R. FERRACINI
*(I.R.C.C, Candiolo (TO), Italy)*

Chemotherapy resistance of cancer cells is the major limitation to this treatment approach. Multidrug resistance (MDR) in many human cancers may be due to the expression of the multidrug transport P-glycoprotein (Pgp). New drugs developed specifically to inhibit Pgp, such as Valspodar (PSC 833—Novartis), may provide clinicians with more potent and specific inhibitors for MDR modulation trials.
We have investigated the cytotoxic effect of doxorubicin (DXR) combined with PSC 833 on cultured human sarcoma cell lines (U-2 OS, U-2/neo8, U-2 OS/DX580, OS/DX35, OS/DX117.1, OS/DX117.2), expressing various levels of Pgp. The major cytotoxic effects were achieved through the continuous presence of PSC 833 in the medium, therefore showing that sustained effective concentration of PSC 833 during DXR administration is needed in order to obtain maximal effect.
We have furthermore evaluated Pgp expression in sporadic canine osteosarcoma, confirming its high incidence. These data let us develop a novel protocol for the treatment of canine osteosarcoma using PSC 833 and DXR. We have evaluated the pharmacokinetic, toxicity and side effects of this combined treatment that resulted active and well tolerated.
Our studies, considered with the previous literature, support a rational to develop a risk-adapted strategy in human patients with Pgp positive cancer at clinical onset that could be treated with combined regimes including DXR and PSC 833, in order to revert multidrug resistance. Since soft tissue sarcomas are shown to express MDR phenotype in a high percentage of cases, while present chemotherapeutic treatments of these tumours need major improvements, we therefore propose to develop a clinical trial using DXR in association with PSC 833 in sarcoma patients whose primary tumours were shown to express detectable levels of Pgp.

**Prognostic value of microvessel density, proliferation and apoptosis in liposarcoma**

E.G. ACHILLES*, S. LASCH*, J. SCHULZ*, J. SCHULTE A.M. ESCH*, M. PEIPER°, W.D. BEECKEN+, O. KISKER‡, X. ROGIERS* & J.R. IZBICKI°
*(From the Departments of °General and *Hepatobiliary Surgery, University Hospital Hamburg, Hamburg, and the +Clinic for Urology, University Hospital Frankfurt/Main, Frankfurt, and the ‡Department of General Surgery, Philipps University Marburg, Marburg Germany)*

*Purpose:* As an adjunct to conventional grading in human liposarcomas, the possible prognostic value of intratumoural microvessel density, rate of tumour cell apoptosis and proliferation was investigated.
*Patients and Methods:* 51 patients (female n = 21, male n = 30) with liposarcoma resected between 1988 and 2000 in our center were included in this study. Tumours were localised in the extremities (n = 28), retroperitoneum (n = 20) and trunk (n = 3). Tumour margins were free (n = 25) or showed residual microscopic (n = 25) or macroscopic (n = 1) disease. Multiple variables for each patient, including age, sex, tumour size, grading and numbers of microvessels, apoptosis and proliferating cells were determined. Immunocytochemistry was performed for semiquantitative analysis of representative paraffin embedded tissue sections. Monoclonal antibodies targeted to platelet/endothelial adhesion molecule (CD-31), proliferating cell nuclear antigen and apoptotic nuclei (Tunel-assay) were utilised. The results were correlated with the postoperative course of these patients.
*Results:* The median survival time was 10.2 years. Multivariate analysis (Cox—regression) revealed no significant correlation between survival and all variables tested.
*Discussion:* These data indicate that in liposarcoma—contrary to carcinoma—microvessel density, tumour cell apoptosis and proliferation are of no prognostic value in the assessment of disease—specific survival. Further studies are currently undertaken to determine the prognostic potential of these variables in a larger series including other types of soft tissue sarcoma.

**C-reactive protein levels in patients with malignant fibrous histiocytomas**

H. NAKANISHI[1], N. ARAKI[1], I. KUDAWARA[2], A. MATSUMINE[2], M. MANO[3], T. UEDA[4] & H. YOSHIKAWA[1]
*([1]Department of Orthopedic Surgery, Osaka Medical Center for Cancer and Cardiovascular Diseases, [2]Department of Orthopedic Surgery, National Osaka Hospital, [3]Department of Pathology, Osaka Medical Center for Cancer and Cardiovascular)*

Paraneoplastic syndrome, for example, anorexia, weight loss, leukemoid reaction, frequently occurs in some advanced cancer patients. However, these phenomena in mesenchymal tumor patients are relatively uncommon except for malignant fibrous histiocytoma (MFH). Since there has been no report concerning the incidence and clinicopathological characteristics of paraneoplastic syndrome in MFH, we measured the serum C-reactive protein (CRP) concentration in MFH patients continually from the first admission, and analyzed its correlation with the clinicopathologic features.
*Methods:* Thirty patients (male/female; 12/18) with primary soft-tissue MFH were studied, who underwent radical surgery between May 1988 and Dec 2000. The mean age was 59 years (range; 43–85). The correlation between the serum CRP levels and the clinicopathologic features (size, depth, grade, metastasis) was analyzed.
*Results:* In twenty-one patients (70%), the preoperative serum CRP level was elevated. The histologic type was predominantly striform-pleomorphic type (19 cases; 90%). In 19 cases (90%) out of the elevated CRP cases, paraneoplastic syndrome such as fever, leukocytosis, anemia, hypoalbuminemia, and hepatic dysfunction developed. When the tumor was removed, the elevated CRP levels subsided into the normal range in all cases. Tumor size and depth were significantly different between two groups ($p<<0.05$, $p<<0.001$, respectively), but not in histological grade and pulmonary metastasis rate. In 2 cases out of 3 relapsed cases, the serum CRP level re-elevated with the tumor regrowth.
*Discussion:* Paraneoplastic syndrome was not an unusual event in MFH, especially in the cases with an elevated serum CRP level. Serum CRP level may be a useful indicator of the disease status and a new tumor marker to detect the relapse of MFH.

**Multidrug resistance in soft tissue sarcomas: downregulation of P-glycoprotein during metastatic progression.**

R. KOMDEUR, W.M. MOLENAAR, N. ZWART, H.J. HOEKSTRA, E.V.D. BERG, W.T.A.V.D. GRAAF
*(Groningen University Hospital, Groningen, The Netherlands)*

Metastatic soft tissue sarcomas (STS) have a response rate of only 20–30% to standard chemotherapy (doxorubicin, ifosfamide), possibly due to the so-called multidrug resistance (MDR). MDR is associated with the over-expression of P-glycoprotein (P-gp), Multidrug Resistance-associated Protein 1 (MRP1) and Lung Resistance-related Protein (LRP). Since it is unknown if metastatic STS are more resistant than their primary counterparts, we studied the MDR-status in paired samples. Samples from 33 chemonaive primary STS and their metastases were collected; 85% of the metastases was unexposed to chemotherapy. Expression was assessed immunohistochemically, using monoclonals C494 (P-gp; Signet Lab), MRPr1 (MRP1; Dr Scheper, Free University Amsterdam) and LRP (LRP; Transduction Lab). Expression was scored as positive (>5% positive tumor cells) or negative. P-gp expression was positive in 29/32 primaries (91%), versus 22/30 metastases (73%); paired analysis revealed significantly fewer P-gp positive metastases: $P<0.05$. MRP1 was positive in 17/30 primaries (57%), versus 19/30 metastases (64%). LRP was positive in 25/32 primaries (78%), versus 26/32 metastases (81%). MRP1 and LRP expression did not significantly differ between primaries and metastases. In conclusion, this selective group (all metastasised) revealed a high expression of P-gp, MRP1 and LRP in the primaries. Remarkably, significantly less metastases were P-gp positive ($P<0.05$). MRP1 and LRP expression in the primaries did not differ from the metastases in this group. These results suggest that metastatic progression does not coincide with upregulation of MDR status in STS.

**GP170, bcl2, MIB1 and TopoII expression in abdominal leiomyosarcoma (ALS) and in round cells sarcoma (RCS). Correlation with biological characteristics, grading and response to chemotherapy (CT).**

A. COMANDONE*, A. BOGLIONE*, E. BERARDENGO#MC, R. BUSSONE", A. BERNARDI#MC, O. DAL CANTON*, C. OLIVA* & P. BERGNOLO*
*(*Medical Oncology, Ospedale Gradenigo, #mCDivision of Pathology and "Surgery, Ospedale SanGiovanni, Torino, Italy)*

ALS account for 5% of soft tissue sarcomas (STS). These are tumours of adult-aged life, more common in women than in men. CT is scarcely active. On the contrary RCS is typical for children under 15, more common in men than women. We compared the expression of GP170, bcl2, MIB1 and TopoII in 23 ALS and 18 RCS paraffin-embedded samples. Results ALS: MIB1+ 56%, TopoII+ 34%, with a significant correlation ($p = 0.03$) to histology. The same markers weren't correlate to grade, bcl2 and GP170 activity. Moreover no correlation to ADM therapy and response was seen in 11/23 pts treated with CT. RCS: all but three were G3 tumours (83%). MIB1 was positive in 100% and TopoII in 94% of the cases ($p<0.001$ to histology). No correlation with TopoII/grade, GP170/histology and bcl2/histology and grade was seen. Only 6/18 pts received CT and statistical considerations cannot be done. In conclusions Immunohistochemically detection of proliferate indexes (MIB1 and TopoII) may add some important information in the prognostic criteria either in ALS or in RCS. Less defined is the role of GP170, bcl2, MIB1 and TopoII to predict response to CT.

**Lung resistance-related protein expression in rhabdomyosarcomas in children versus adults**

J.W. KLUNDER[1]; R. KOMDEUR[2]; W.T.A. VAN DER GRAAF[2]; H.J. HOEKSTRA[3]; E. VAN DEN BERG[4] & W.M. MOLENAAR[1]
*(Depts. of Pathol.[1], Int. Med.[2], Surg.[3] and Med. Genet.[4], Univ. of Groningen, Groningen, The Netherlands)*

*Purpose:* The prognosis of patients with a rhabdomyosarcoma, treated with chemotherapy, seems better in children compared to adults. This could be due to overexpression of Lung Resistance-related Protein (LRP).
*Material and methods:* Primary, chemonaive rhabdomyosarcomas of 24 children (24 embryonal, including 2 botryoid) and 15 adults (9 embryonal, 3 alveolar and 3 pleomorphic) were assessed immunohistochemically using an LRP antibody (Transduction Laboratories, Los Angeles, CA). Patients were considered adult when the age at diagnosis was 16 years or higher, the samples were considered positive if ≥5% of the tumor cells was immunoreactive.
*Results:* Although not significant ($p<0,1$), adults were found to have a higher expression of LRP, i.e. 73% of the tumours from adults and 46% of those from the children. LRP immunoreactivity was especially prominent in better differentiated cells. Among the embryonal tumours 7 of 9 (78%) tumours from adults were positive as compared to 11 of the 24 (46%) in children. All three pleomorphic and 1 of 3 alveolar tumours, i.e. types of rhabdomyosarcoma with a poorer prognosis, were positive.
*Discussion:* There is a difference in LRP expression in rhabdomyosarcomas between adults and children. Whether this may explain the worse outcome of adults with rhabdomyosarcomas as compared to children needs further investigation.

**Multidrug resistance protein expression in rhabdomyosarcomas before and after chemotherapy**

W.M. MOLENAAR[1], J.W. KLUNDER[1]; R. KOMDEUR[2]; H.J. HOEKSTRA[3] & V.D.E. BERG[4]; W.T.A. VAN DER GRAAF[2]
*(Depts. of Pathol.[1], Int. Med.[2], Surg.[3] and Med. Genet.[4], Univ. of Groningen, Groningen, The Netherlands)*

*Purpose:* Rhabdomyosarcomas generally respond fairly well to chemotherapy. The residual lesions often show morphologic differentiation, presumably caused by selective insensitivity of differentiated tumor cells to chemotherapy. Insensitivity may be related to multidrug resistance, associated with overexpression of P-glycoprotein (P-gp), Multidrug Resistance Protein 1 (MRP1) and Lung Resistance Protein (LRP). Therefore, pairs of rhabdomyosarcomas before and after chemotherapy were compared.
*Material:* Tissue of 10 primary rhabdomyosarcomas from 5 adult and 5 pediatric patients and of 8 corresponding residual tumours and 2 metastases was assessed immunohistochemically for P-gp, MRP and LRP. The samples were scored semiquantitatively.
*Results:* All specimens after chemotherapy showed clear differentiation as compared to their primaries. All except one primary tumours expressed P-gp and all but three MRP. LRP was much less extensive and absent or less than 5% in 4 cases. In all but one case each, P-gp and MRP expression after chemotherapy was similar or even less than before. In contrast, LRP (strongly) increased after chemotherapy in 7 cases. Moreover, both in primary and residual tumours, LRP expression was most prominent in the most differentiated tumor cells.
*Discussion:* The morphologic differentiation of rhabdomyosarcomas under the influence of chemotherapy may be related to selection of LRP expressing, differentiated tumor cells.

**Detection of SYT-SSX fusion gene in peripheral blood from a patient with synovial sarcoma**

A. MYOUI, N. HASHIMOTO, N. ARAKI, T. ASAI, H. SONOBE, S. HIROTA & H. YOSHIKAWA
*(Department of Orthopedic Surgery, Osaka University Medical School, 2–2 Yamada-oka, Suita 565–0871, JAPAN)*

*Purpose:* Recent molecular analyses revealed that a specific translocation, t(X;18)(p11.2;q11.2) in synovial sarcoma (SS) induces a fusion gene, SYT-SSX. The purpose of this study is to establish the sensitive method to detect circulating SS cells by RT-PCR.

*Patient:* A 22-year-old woman with poorly-differentiated SS in the right thigh was presented to our hospital. She was initially treated with wide excision of the tumor alone because she was in 27th week of pregnancy when the diagnosis was made. However, multiple lung metastases developed six weeks after primary tumor excision, and intensive chemotherapy was given after delivery.
*Methods:* Peripheral blood samples were drawn before biopsy, two months after primary tumor excision when multiple lung metastases were apparent on chest X-rays, and after first cycle of chemotherapy. mRNA was extracted from the blood samples. After RT-PCR with a primer set that is specific for SYT-SSX fusion gene transcripts, the PCR product was subjected to nested PCR with second set of primers that yields 212-bp product from the first 585-bp PCR product.
*Results and discussions:* The SYT-SSX fusion gene transcript was detected by nested PCR in the peripheral blood collected prior to biopsy, but not after the primary tumor excision. Six weeks after the tumor resection, multiple lung metastases developed. This is the first reported case in which tumor cells were detected by nested PCR in the peripheral blood of a SS patient, and it is suggested that monitoring of the circulating tumor cells may be a prognostic indicator for SS patients.

**C-erbB-4 expression in limb STS: correlation with neoadjuvant chemotherapy results.**

O. MERIMSKY, J. ISSAKOV, J. BICKELS, Y. KOLLENDER, G. FLUSSER, S. VJACHESLAV, I. SCHWARTZ, M. INBAR & I. MELLER
*(O. Merimsky, MD, Dept. of Oncology, The Tel-Aviv Sourasky Medical Center, 6, Weizman Str. Tel-Aviv 64239, Israel)*

*Purpose:* ErbB-4 is a recently described member of the epidermal growth factor receptor (EGFR) family. Relatively little is known about the expression of erbB-4 in human tumours. In the present study we assessed the possible role of c-erbB-4 expression product as a tissue marker for STS, and its correlation with the response to chemotherapy. *Patients*: The histological specimen of 29 patients with STS of a limb who had received preoperative doxorubicin-based chemotherapy were studied. The extent of tumor necrosis was evaluated histologically. Paraffin blocks of preoperative incisional biopsy were available for immune staining (avidin-biotin-peroxidase technique) from 29 patients, and blocks of the surgical specimen after pre-operative chemotherapy were available from 27. *Results*: The objective response rate to preoperative chemotherapy was 34%. Wide resection of the tumor was feasible in 12 patients, marginal resection in 14 cases, amputation in 2 patients with disease progression, and no surgery in one case. The tumor necrosis was above 90% in 9 patients, 60–90% in 12, and less than 60% in 7 patients. It was found that an increase in C-erbB-4 expression was more common in cases with no response to chemotherapy, while no change of or decrease in C-erbB-4 was more common in responsive tumours ($p = 0.004$). No correlation could be found between the degree of necrosis or the chemotherapeutic regimen and the change in expression of c-erbB-4. The median DFS was longer for patients with a decrease or no change in expression of C-erbB-4 than for patients with increased expression.
*Discussion:* It is believed that post chemotherapy new expression or no down-regulation of the erbB-4 molecule represents tumor aggressiveness and increased capability of growth and spread.

**Expression of CD44 in Ewing's sarcoma**

A. AFENYI-ANNAN, M.D., M.A. RUBIN & A.F.G. PAULINO, M.D.
*(University of Michagan Hospitals, Ann Arbor, Michigan, USA)*

*Purpose:* CD44, a transmembrane glycoprotein, has been implicated in tumourigenesis and metastasis in carcinoma. It may potentially be a diagnostic/prognostic marker and/or target for cancer therapy. Expression of CD44 has been described in soft tissue sarcomas but not in Ewing's sarcoma. We studied the use of CD44, a hyaluronidase receptor, as a marker for Ewing's sarcoma and correlated expression with clinical outcome.
*Patients:* Surgical pathology and clinical follow-up from 55 patients with Ewing's sarcoma at our institution between 1987–2000 was retrospectively reviewed. Cases with insufficient tumor material were excluded from the study.
*Methods:* Microscopic slides from 67 cases of Ewing's sarcoma were stained with monoclonal antibodies for MIC-2 (CD99) and standard CD44 using routine immunohistochemical (IHC) techniques. The majority of cases were stained in triplicate from different tumor samples.
*Results and Discussion:* The presence of Ewing's sarcoma was confirmed by MIC-2 (CD99) positivity in 98.5% (66/67) of cases. CD44 expression was seen in 88% (59/67) of cases with an intense, membranous staining pattern. CD44 expression did not correlate with age, sex, and tumor site. Furthermore, there was no statistical significance between patient survival and histologic response to preoperative chemotherapy and CD44 expression.
IHC staining for MIC-2 (CD99) remains the gold standard in confirming the diagnosis of Ewing's sarcoma. However, Ewing's sarcoma clearly expresses the standard CD44 antigen. Further studies are needed to determine how CD44 and/or its variants affect growth, metastasis, and prognosis in Ewing's sarcoma.

**Post-chemotherapy tumor necrosis in Ewing's sarcoma: the Huvos and Picci Grading Systems Revisited**

A. AFENYI-ANNAN, M.A. RUBIN & A.F.G. PAULINO
*(University of Michigan Hospitals, Ann Arbor, Michigan, USA)*

*Purpose:* This study compares the two common grading schemes used to assess tumor necrosis in patients with Ewing's sarcoma treated by a combination of pre-operative chemotherapy and surgical resection and relates these systems with patient outcome. The chemotherapeutic response is measured based on necrosis as a percentage of tumor volume (Huvos system) or on defined microscopic guidelines, not volume dependent (Picci system). The ability to predict patient outcome based on the post-chemotherapy tumor necrosis in resection specimens is well established in patients with osteosarcoma but less so in Ewing's sarcoma.
*Patients:* Thirty patients with Ewing's sarcoma treated with chemotherapy followed by surgical resection between 1987 and 2000 were evaluated. The median age was 14.5 years (range, 3 to 55 years). The male to female ratio was 1.5:1. Tumor location included both skeletal (24) and extra-skeletal (6) sites.
*Methods:* Microscopic slides were reviewed and evaluated based on the two systems of post-therapy tumor necrosis. Results were stratified into Grades I-IV (Huvos system) and Grades I-III (Picci system) and compared with patient outcome.
*Results and Discussion:* Post-chemotherapy tumor necrosis in Ewing's sarcoma was not an independent prognostic factor in our series. Survival rates for each grade between systems were comparable (i.e. Huvos grades III-IV and Picci grade III). No statistical significance between assessment schemes was identified. Neither system of assessing response to therapy was better at predicting patient outcome.

**An analysis of late events and outcome of localised ewing's sarcoma of bone in patients with a minimum of 10 years follow-up**

A. ABUDU, R.J. GRIMER, S.R. CARTER, R.M. TILLMAN, P.B. PYNSENT, A.M. DAVIES, D.C. MANGHAM & D. SPOONER
*(Royal Orthopaedic Hospital Birmingham, UK)*

*Purpose:* To study the long-term outcome, risk of late events and risk factors for survival and local control in patients with Ewing's sarcoma of bone without identifiable metastases at diagnosis.
*Patients/Subjects:* 95 patients with localized primary Ewing's sarcoma of bone with minimum of 10 years follow-up and treated with the same protocol were studied. All received neoadjuvant chemotherapy according to UKCCSG protocol. Local treatment was surgical excision in 60, surgical excision and radiotherapy in 20, and radiotherapy in 15 patients.
*Results:* 10-year overall and metastases free survival was 60% and 55% respectively. Local recurrence occurred in 10 patients and was dependent on location of tumour and surgical margins. There was a significant relationship between local recurrence and metastasis. 14% of metastases occurred after 5 years follow-up and was seen up to 10 years from diagnosis but not beyond.
Survival was dependent on age at diagnosis, post-chemotherapy necrosis and development of local recurrence on multivariate analysis.
*Discussion:* Development of local recurrence was ominous. Surgical treatment of tumours instead of radiotherapy had a significant influence on outcome in patients with extremity tumours but not in those with pelvic tumours. Patients with Ewing's sarcoma remain at risk of disease recurrence in the first 10 years of diagnosis and are probably cured of disease after more than 10 years of continuous disease free period. The risk of radiation-induced sarcoma in patients managed on a modern radiotherapy protocol is low in the short term.

**Concurrent conformal radiotherapy (CRT) and chemotherapy in patients with primitive neuroendocrine tumours (PNETs), ewings sarcoma and leiomyosarcoma.**

J. ABRAHAM; C. COLES; N. BURNET & H.M. EARL
*(Addenbrookes Hospital, Cambridge)*

*Purpose:* Does conformal radiotherapy (CRT) reduce acute toxicity and prevent treatment delays for both modalities when using concomitant chemoradiotherapy.
*Method:* 6patients (3Ewings, 2PNETs and 1Leiomyosarcoma) treated between 11/96–05/00. Median age 27 (range 16–60), male:female ratio 5:1. Locations: Pelvis(2); Chest wall(2); Femur(1); Thigh(1). 1patient had lung metastases at diagnosis.
Chemotherapy regimens: EVAIA (2/2PNETs) and VAIA (1/3Ewings) from EICESS '92 study; VIDE/VIA (2/3Ewings) standard arm of Euro-Ewings '99 protocol; VIA (1Leiomyosarcoma). 2 or more cycles of chemotherapy were administered concurrently with CRT. CRT was given as per EICESS '92 protocol, except the Leiomyosarcoma patient. CRT reduces volume of normal tissue irradiated including bone marrow. Case notes were reviewed for treatment delays and acute toxicities. Additional toxicity data were available from standardised flow charts (4/6patients).
*Results:* 4/6patients achieved all scheduled treatments without delay. Neutropenic sepsis delayed 1 chemotherapy cycle (1/6patients) and the start of CRT (1/6patients). Grade3 toxicities included oral mucositis (2/6patients), vomiting (1/6patients), and neutropenic sepsis (3/6patients). Grade4 neutropenic sepsis (1/6patients). There were no acute radiotherapy toxicities. 5/6patients required 10–25% dose reductions and GCSF.
*Discussion:* Our experience suggests CRT facilitates scheduled delivery of chemoradiotherapy and reduces acute toxicities compared with conventional radiotherapy. 4/6patients achieved all scheduled treatments. Standardised forms improved toxicity assessment accuracy. Optimal treatment schedules allow dose-dense chemotherapy and potentially increased tumour kill by combined chemoradiotherapy. We hope this will give better overall survival and increased local control. We plan a 20patients phase II pilot study of concurrent chemotherapy/CRT with primary endpoints of local recurrence and overall survival.

**Peripheral primitive neuroectodermal tumor/ewing's sarcoma of the meninges. A report of two cases.**

R. SCIOT, C. GIANINI* & A.P. DEI TOS*
*(Department of Neuropathology, University Hospital K.U. Leuven, Leuven, Belgium, * Department of Pathology, Regional Hospital Treviso, Italy)*

*Purpose:* To report on the occurrence of a primary peripheral primitive neuroectodermal (PNET) in the meninges of two patients.
*Patients:* The first patient was 17 year old boy with a frontal, dura based tumor. The second patient, a 12 year old boy, presented with a dura attached parietal parasagittal mass. There was no bone involvement and no primary tumor/metastases could be detected outside the CNS. Both tumors were resected and both patients received chemo-, and radiotherapy.
*Results:* Compact nests of uniform 'small blue round cells' were seen in both lesions. On immunohistochemistry, a strong CD99 and vimentin expression was seen. Synaptophysin, neurofilament, Epithelial Membrane Antigen, cytokeratin, and GFAP were negative. A t(11;22)(q24;q12) could be demonstrated with RT-PCR in case 1 while FISH analysis showed a rearrangement of the EWS gene on 22q12 in case 2. The CD99 expression, as well as the molecular findings indicate that both lesions represent a peripheral PNET.
*Discussion:* Meningeal peripheral PNET is extremely rare as evidenced by the presence of only one reported case with proven t(11;22)(q24;q12). The result of this translocation is a fusion of the EWS gene (22q12) with a truncated transcription factor FLI1(11q24), resulting in an oncogenic conversion of the EWS gene. These molecular findings clearly differ from the central PNET, in which an isochromosome 17(q) is characteristically found, mainly in medulloblastoma. In addition to immunohistochemistry, detection of the t(11;22)(q24;q12) is extremely useful to discriminate peripheral PNET from a central PNET, which is much more common in this location.

**Activation of peroxisome proliferation activated receptor induces survival of human osteosarcoma cells.**

E. LUCARELLI, L. SANGIORGI, V. MAINI, J. WARZECHA & P. PICCI
*(Istituto Ortopedici, Bologna, Italy)*

*Purpose:* Because activation of PPAR has been shown to promote apoptosis in several tumor cell lines, in this study we investigated whether PPARγ activation, stimulated by torglitazone (TZD), induced apoptosis in a human osteosarcoma (OS) cell lines that expresses PPAR.
*Subject:* PPARγ is a member of the nuclear receptor superfamily. PPAR_ binds to the promoter region of a target gene as an heterodimer with the retinoid X receptors (RXR). Several natural and synthetic ligands bind to PPARγ such as the natural occurring 15-deoxyδ 12, 14 prostaglandin J2 and fatty acids derivatives and the synthetic class of drug thiazolidinediones (TZDs).
*Results:* In our experiments, TZD treatment never induced apoptosis of OS cells; on the contrary, TZD increased cell number, based on MTT proliferation assay. Remarkably, the TZD-induced increase in cell number depended on a decrease of apoptosis that naturally occurred in the culture and was not due to an increased cell proliferation rate. TZD prevented apoptosis also when induced by staurosporin. The TZD-mediated survival effect correlated with the activation of Akt, a well known mediator of survival stimuli.

*Discussion:* Our results suggest that PPARγ activation may be a key element that inhibits cell death and promotes tumor growth. Results from this study caution against the use of PPAR_chemical agonist on patients with OS and suggest that PPARγ activation by natural ligands may be a key step in OS development.
